# A proteomic profile of the healthy human placenta

**DOI:** 10.1186/s12014-022-09388-4

**Published:** 2023-01-02

**Authors:** Samprikta Manna, Julia Scheel, Aisling Noone, Colm J. McElwain, Caitriona Scaife, Shailendra Gupta, Jane English, Cathal McCarthy, Fergus P. McCarthy

**Affiliations:** 1 Department of Obstetrics and Gynaecology, Cork University Maternity Hospital, University College Cork, Cork, Ireland; 2 Department of Pharmacology and Therapeutics, Western Gateway Building, University College Cork, Cork, Ireland; 3 INFANT Research Centre, Cork, Ireland; 4 Department of Anatomy and Neuroscience, Western Gateway Building, University College Cork, Cork, Ireland; 5 Dept. of Systems Biology and Bioinformatics, University of Rostock, 18057 Rostock, Germany; 6 UCD Conway Institute for Biomolecular and Biomedical Research, University College Dublin, Dublin, Ireland

**Keywords:** Placenta, Proteomics, Bioinformatics, Network analysis, Disease map, Systems biology

## Abstract

**Background:**

The placenta remains one of the least studied organs within the human body. Yet, placental dysfunction has been associated with various pregnancy complications leading to both maternal and fetal death and long-term health consequences. The aim of this study was to characterise the protein networks of healthy term placental sub-anatomical regions using label free quantification mass spectrometry.

**Methods:**

Three healthy placentae were sampled at five sample sites and each biopsy was dissected into maternal-, middle-, and fetal- sub-anatomical regions. Quadrupole-orbitrap mass spectrometer was used in data dependant analysis mode to identify 1859 unique proteins before detailed differential expression between regions.

**Results:**

Protein profiling identified 1081, 1086, and 1101 proteins in maternal, middle, and fetal sub-anatomical regions respectively. Differentially expressed proteins were identified considering the effect between sample site location and sub-anatomical region on protein expression. Of these, 374 differentially expressed proteins (Two-way ANOVA adjusted p-value < 0.05, HSD Tukey adjusted p-value 0.05) were identified between sample site locations and sub-anatomical regions. The placenta specific disease map NaviCenta (https://www.sbi.uni-rostock.de/minerva/index.xhtml?id=NaviCenta) was used to focus functional analysis results to the placenta specific context. Subsequently, functional analysis with a focus on senescence, and mitochondrial function were performed. Significant differences were observed between sub-anatomical regions in protein intensity and composition. A decrease in anti-senescent proteins within the maternal sub-anatomical region, and an increase in proteins associated with a switch from ATP to fatty acid consumption as a source of energy between middle and fetal sub-anatomical regions were observed.

**Conclusion:**

These results suggest that normal proteomic variations exist within the anatomical structure of the placenta, thus recommending serial sectioning methodology for consistent placental research.

**Supplementary Information:**

The online version contains supplementary material available at 10.1186/s12014-022-09388-4.

## Background

The placenta is a complex organ playing a critical role in the development and preservation of a healthy pregnancy. It is appropriately defined by Harland Mossman as “The normal mammalian placenta is an apposition or fusion of the fetal membranes to the uterine mucosa for physiological exchange” [1, 2]. The placenta is a dynamic organ undergoing rapid growth, and dramatic molecular and histological rearrangements along gestational stages, in order to adapt to fetal requirements [3].

Nevertheless, the placenta remains the least studied organ in the human body, whose structural and functional composition have possible implications for long term maternal and child health outcomes [4, 5]. A healthy placenta at term is a disc shaped organ around 22 cm in diameter and weighing approximately 500 g [2]. Although the placental is a fetal organ, its anatomical regions can be loosely termed as: Maternal and Fetal, based on its proximity to foetus [6, 7]. This multifaceted transient organ is characterised by different anatomical structures; the chorionic plate with umbilical cord attached, at the fetal side (fetal sub-anatomical region), the basal plate which borders the maternal endometrium (maternal sub-anatomical region) and the intermediate region connecting the basal plate and chorionic plate (middle sub-anatomical region) [8, 9]. Placental villi, the core structural unit are classified as stem villi which connect to the chorionic plate with limited exchange functionality; intermediate villi classified as mature or immature consisting of cytotrophoblast subtypes with differing functional roles and the syncytiotrophoblasts layer which acts as principal transfer site especially in first and second trimester. As pregnancy progresses the terminal villi which are completely formed at 20 weeks’ gestation, are the dominant morphological feature with syncytiotrophoblasts being immersed in maternal blood facilitating its functional role in nutrient and gaseous exchange [6, 10]. The placenta ages gradually as the pregnancy proceeds. At 40 weeks of gestation, the placenta is at its verge of decline, exhibiting morphological and physiological senescence [11]. However, in presence of undesirable oxidative stress, placental aging may be accelerated in certain pregnancy conditions such as preeclampsia (PE), intrauterine growth restriction (IUGR) and stillbirth, characterized by both mitochondrial dysfunction and premature cellular senescence [12, 13].

The placental proteome has been used to further our understanding of the molecular mechanisms involved in development of the placenta and in the pathophysiology of placental disorders [3]. Furthermore, this technology has been used in clinical research to aid in the discovery of protein or metabolite biomarkers to incorporate as a diagnostic tool for clinical utility [14]. Proteomics has been widely used in pregnancy complication such as PE and IUGR, by using novel methods in specific trimester of pregnancies, for diagnosis, and understanding pathophysiological mechanism to improve maternal and neonatal health outcomes [15].

Uniformity of placental sampling has been lacking in research with some studies focusing on single placental sampling sites which may not always be representative of the global signature of this large heterogenous metabolic organ. Standardization of placental tissue sampling can improve the quality of placental research, and facilitate sharing of samples between groups, allowing larger datasets to be generated [16]. Burton et al., summarized the factors such as methodological flaws including sampling and processing samples that might be limiting placental research [17]. The aim of this research was to determine the proteomic signature of a healthy term placenta, while investigating proteomic composition and molecular pathways enriched across the placental sub-anatomical regions.

## Methods

### Patient recruitment and sample collection

Subjects were recruited as part of the COMRADES Study, a non-interventional cohort study of nulliparous singleton pregnancies with the aim of characterizing placental premature senescence in preeclampsia (PE) and Intra-uterine growth restriction (IUGR). The COMRADES study was conducted according to the guidelines laid down in the Declaration of Helsinki, and all the procedures were approved by the Clinical Research Ethics Committee of the Cork Teaching (ECM4 (ff) 04/12/18). All women provided written informed consent to take part in the study. Three normotensive, healthy pregnant participants with no obstetric complications were recruited as a part of the COMRADES Study between February and July 2020. Placental samples were collected from nulliparous, singleton pregnancies undergoing Caesarean section for non- obstetric indications such as breech presentation with consent from the participants. Placental sampling was completed within 20 min of delivery, snap frozen in liquid nitrogen and stored at -80 °C for long term storage. Placental sectioning was performed in accordance with the standard protocol described by Burton et al. [16].

Tissue samples [2–4 cm in diameter] were isolated from 5 different sample sites across the placenta. We defined the sub-anatomical regions of the placenta as: maternal, middle, and fetal to evaluate the basal plate, chorionic plate and the middle part connecting the two surfaces of the placenta. Sectioning was performed by keeping close to the fetal membrane from the chorionic plate, the maternal part was sectioned closer to the basal membrane and the middle region was selected from between maternal and fetal sections (Additional file 1: Figure S1). Fetal membranes were not included in the sectioning [16].

### Protein extraction and quantification

Processing of placental tissue for proteomics analysis was based on previously established laboratory protocols [18]. Placental tissue (50 mg) was homogenized using TissueLyser II (Qiagen, Germany) and protein isolated) using Tetraethylammonium bicarbonate (TEAB) buffer (Sigma-Aldrich, Ireland) in presence of Phosphatase inhibitor (PhosSTOP™, Roche, Ireland) and Protease Inhibitor (Roche, Ireland) Mechanical tissue homogenization. Protein quantification was assessed with BCA Assay (Pierce™ Thermo Fisher Scientific, USA) and protein volume adjusted to 50 µg/50 µl concentration with MS grade dH_2_O (Additional file 2).

### Protein solubilisation and desalination

Protein solubilisation and desalination process has been previously described [18]. Protein enzymatic digestion was performed using Trypsin (Promega, MyBio, Ireland) with the aid of Rapigest (1%) solution (Waters, Ireland). To aid with Trypsin (Promega – details) 50 mM Tris(2-carboxyethyl) phosphine hydrochloride (TCEP) (Sigma-Aldrich, Ireland) solution was added to the denatured proteins and samples reduced for 60 min at 60 °C, followed by addition of 200 mM Indole-3-acetic acid (IAA) solution (Sigma-Aldrich, Ireland) to alkylate proteins, which were incubated overnight at 37 °C with 1:100 sequential grade trypsin according to manufacturer’s instruction followed by addition of 1% Formic acid was used to stop digestion. Precipitation of Rapigest was done by centrifuging samples at 13,000 rpm for 10 min and supernatants collected and air dried on MiVac Quattro sample concentrator (Fisher Scientific, Sweden) followed by resuspension in 0.5% Formic Acid (FA) (Sigma-Aldrich, Ireland). Desalting and sample clean-up was performed using Zip-Tip columns with 0.6 µl binding volume C18 resin (Sigma-Aldrich, Ireland). For maximum binding of protein to resin present on zip-tip column, the tips are equilibrated using 0.1% Trifluoroacetic acid (TFA) in H2O; followed by washing and binding of protein, and finally the protein are eluted into 10 µl elution solution composed of 50% ACN in 0.1% TFA. The process was repeated to achieve 1 µg/µl concentration. The samples are then re-dried on MiVac Quattro sample concentrator (Fisher Scientific, Sweden) and all samples were resuspended in 0.1% FA to 1 µg/µl volume for LC–MS/MS analysis. Equal aliquots of digested protein in the experiment were pooled into one sample for internal quality control (QC) standard which was injected three times at the beginning of the MS study and throughout the experiment every 10 to condition the column and monitor the MS performance. LC–MS/MS analysis was performed in the Proteomics Core in the Conway Institute of Biomolecular and Biomedical Research, UCD, Dublin.

### Mass spectrometry (MS) sample preparation

Proteomics samples were enzymatically digested with Trypsin (Promega sequencing grade) according to manufacturer’s instruction and in the presence of Rapigest SF (Waters). Briefly, samples were first reduced with Tris(2-carboxyethyl) phosphine hydrochloride (TCEP) at 60 °C for 60 min, and alkylated with Iodoacetamide (IAA, final concentration y mM) for 30 min in the dark, prior to the addition of trypsin in a ratio of enzyme to protein of 1:100 (w/w). The samples were incubated overnight at 37 °C followed by addition of 1% Formic acid the next morning to stop digestion and precipitate the Rapigest. Removal of Rapigest by-products was performed by centrifugation of samples at 13000 rpm for 10 min and supernatants collected and dried under vacuum with a MiVac Quattro sample concentrator (Fisher Scientific, Sweden). The dried digests were resuspended in 0.5% Formic Acid (FA) (Sigma-Aldrich, Ireland) prior to de-salting with C18 ZipTips (Millipore). Finally, the de-salted digests were resuspended in 0.1% FA for LC–MS/MS analysis. Equal aliquots of digested protein from each sample in the experiment were pooled into a single sample for an internal quality control (QC) standard which was injected three times at the beginning of the MS study and throughout the experiment every 10 samples, and to condition the column and monitor the MS performance. LC–MS/MS analysis was performed in the Proteomics Core in the Conway Institute of Biomolecular and Biomedical Research, UCD, Dublin.

### Mass spectrometry

All samples were injected in a randomized sequence combining Internal standards and samples for discovery of sub anatomical region proteomic profiles. 5 μl of each sample was injected in duplicate onto the Thermo Scientific Q-Exactive® connected to a Dionex Ultimate 3000 High-performance Liquid Chromatography (HPLC) system. The digests were loaded onto a fused silica emitter (75 μm ID), pulled using a laser puller (Sutter Instruments P2000) and packed with UChrom C18 (1.8um) reverse phase media (nanoLCMS Solutions LCC). Peptides were separated using an increasing acetonitrile gradient (250nL/min) for 60 min [18]. The mass spectrometer was operated in data dependent analysis (DDA) mode on a TopN 12 mode with setting: mass range 300-1600Th; resolution for MS1 scan 70,000; AGC target 3e6; resolution for MS2 scan 17,500; AGC target 2e4; charge exclusion unassigned, 1; dynamic exclusion 40 s.

### Bioinformatics and data pre-processing

Raw data obtained from LC–MS/MS runs were searched against the Uniprot Human reference proteome UP000005640 (downloaded 24–05-2021) with MaxQuant (version 1.6.17) using default settings and additionally including the ‘Match Between Runs’ and ‘Label free quantification (LFQ)’ options. The ProteinGroups.txt output file obtained from the MaxQuant analysis was processed in Perseus (version 1.6.15.0). Data was filtered to remove possible contaminants. Samples were categorised by sub-anatomical region. LFQ intensity data was log2 transformed, and proteins not identified in all biological replicates were removed. After rigorous filtering to assure reproducibility, including filtering for valid values in at least 100% of biological replicates, 70% valid values of samples per sample site, and 70% valid values of samples per sub-anatomical region, these proteomic differences did not persist. Furthermore, proteins absent in 70% of samples in at least one sub-anatomical region/sample site were removed. Missing values were replaced by values from the normal distribution.

### Statistical analysis

Statistical analysis was conducted using the built-in statistical tests in Perseus. To analyse the effect of sub-anatomical region and sample site on protein expression a two-way ANOVA was performed. Two-way ANOVA models allow accounting for more than one known source of variation. In this study “sub-anatomical region” and “sample site” were considered independent variables and LFQ protein intensity was the dependent variable (https://www.ncbi.nlm.nih.gov/pmc/articles/PMC2528956/pdf/nihms60473.pdf). Simple main effects were analysed using simple ANOVA and Tukey’s HSD tests. The results were corrected for false discovery (p < 0.05) within Perseus [19].

### Pathway analysis

Functional analysis of differentially overrepresented gene ontology (GO) terms and pathways associated with differentially expressed proteins, was performed using Cytoscape v 3.9.1 ClueGO plugin [20]. GO and pathway term overrepresentation results were corrected for false discovery using Bonferroni step down correction (p < 0.05) and considered significantly overrepresented with an adjusted p value < 0.5.

### Placenta specific characteristics

The term disease map was defined as “a comprehensive, knowledge-based representation of disease mechanisms” [21, 22]. The NaviCenta consists of human and machine-readable representations of processes involved in healthy and dysfunctional placentae in SBGN process description and activity flow (https://www.sbi.uni-rostock.de/minerva/index.xhtml?id=NaviCenta). Phenotype predictions based on user defined data uploaded onto the NaviCenta were performed with plugins [23]. The uploaded information consisted of HGNC identifiers for the genes of all identified proteins, their difference score as calculated in Perseus, and adjusted p values.

### Mitochondria and senescence specific function analysis

Identified proteins that map to mitochondrial function were filtered using or the products of genes associated with mitochondrial functions incorporating information from the Human Protein Atlas mitochondrial sub-cellular location (www.proteinatlas.org) [24], MitoXplorer human interactome [25], and MitoCarta3.0 [26]. The overlap between mitochondrial protein and gene databases, proteins identified in maternal, middle, and fetal regions were visualized using InteractiVenn [27]. Separately, DEPs were filtered to exclusively contain proteins that map to senescence associated molecular process. Senescence associated proteins and genes were identified from the Human Ageing Genomic Resource College Build 20 [28], Cell Senescence Gene Database CSGene [29], and the NCBI gene database with search key “senescence” (https://www.ncbi.nlm.nih.gov/gene/?term=senescence, accessed October 2021). GO biological process terms were visualized using the R Version 4.0.5 and R package GOPlot [30].

## Results

### Study demographics

Patient demographic and clinical information can be found in Table 1. Each placenta had five sample sites (A-E) taken across the placenta. These samples were then separated into three sub-anatomical regions: fetal, middle, and maternal (Additional file 1: Figure S1; resulting in a total number of 45 samples. Sub-anatomical regions were confirmed histologically (Additional file 1: Figure S1).

**Table 1:** Patient demographics and clinical parameters of recruited women

Characteristics	Mean ± SD
Maternal age (years)	34.6 ± 2.5
BMI (kg/m^2^)	23.6 ± 4.1
Parity	0
Gestational age (days)	270.7 ± 5.5
Systolic BP (mm Hg)	122.0 ± 7.5
Diastolic BP (mm Hg)	74.7 ± 5.5
Fetal weight (kg)	3.3 ± 0.2
Fetal individualized customised centile	52.9 ± 5.4
Maternal smoking/tobacco habit	no

### Proteome profile shows variation across placental regions and sample sites

**Fig. 1:**
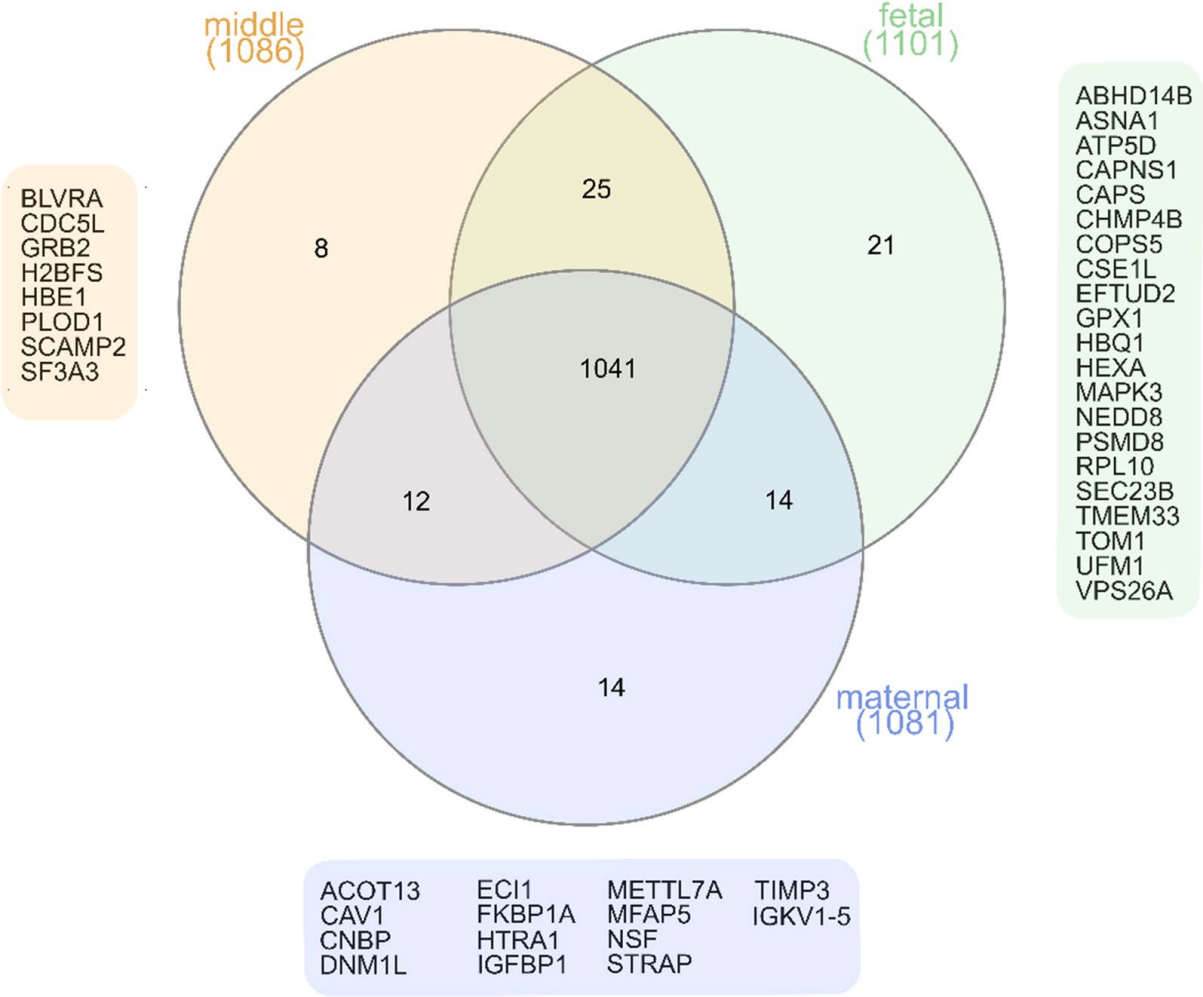
Venn diagram of identified proteins in middle, fetal, and maternal regions. Proteins identified in exclusively one sub-anatomical region are listed next their respective sub-anatomical region

In total 1081, 1086, and 1101 proteins were identified in maternal, middle, and fetal sub-anatomical regions, respectively. We identified 14, 8, and 21 proteins as exclusive to the maternal, middle, or fetal sub-anatomical regions, respectively (Fig. 1).

We analyzed fifteen individual samples were available. Contaminants were removed from further analysis.

EnrichR Cell Marker Database [31] cell type prediction identified only minor differences between sub-anatomical regions (Additional file 1: Figure S2). Although placental tissues were not specifically implicated based on cell markers, most enriched cell types indicate fetal tissues, such as monocytes from fetal kidney, natural killer T (NKT) cells from fetal kidney, mitotic fetal germ cells and other phenotypically similar cell types, which is in line with the placenta being a fetal organ.

Proteomic differences were also found between sample sites, with 520 unique proteins identified in sample site E (Additional file 1: Figure S3). ClueGO functional analysis showed that in particular proteins aligning with GO terms of membrane organization, protein-containing complex and subunit organization, vesicle-mediated transport, organo-nitrogen compound biosynthetic process, and translation initiation factor activity are overrepresented (Additional file 1: Figure S4). All unique proteins to sample site E but SNRPD2, SPP2, EPS8-l1, and ACAA2 were filtered out for the analysis of both sub-anatomical region and sample site considering biological replicates.

### The placental proteome varies with sub-anatomical region

**Fig. 2:**
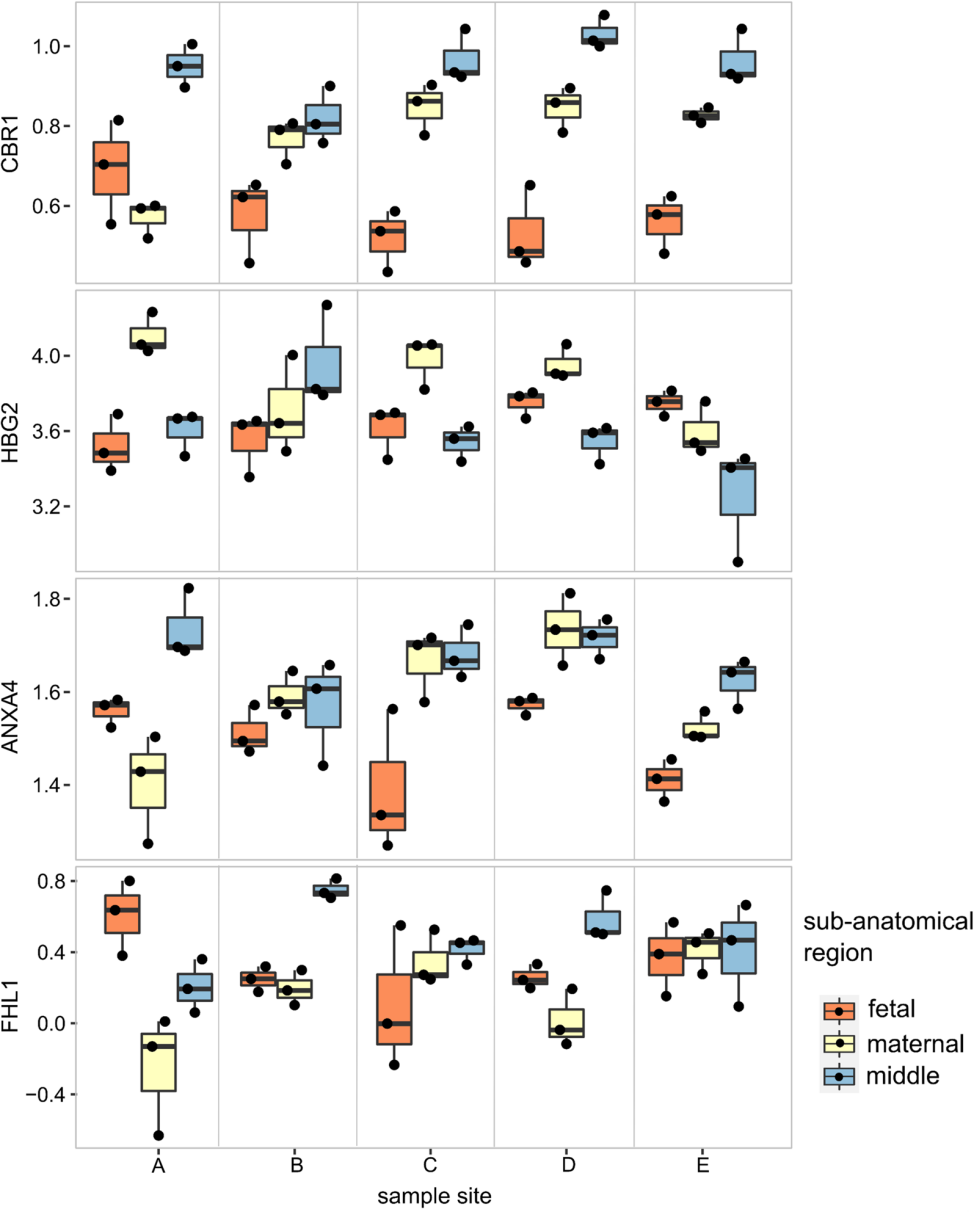
The top four proteins FHL1, ANXA4, HBG2, CBR1 show the strongest interaction between protein content in sub-anatomic regions and sample sites. Interaction was defined as two-way ANOVA adj p val < 0.05. Samples were sorted according to sample site and colour coded according to sub-anatomical region

Two-way ANOVA considering both sub-anatomical region and sample site revealed 374 out of 1038 DEPs (adjusted p-value < 0.05) between sub-anatomical regions. Strong interaction between the effect of sample site and sub-anatomical region on LFQ protein intensity was indicated (p-value interaction < 0.05) for some proteins. The intensity of biological replicates of four proteins (CBR1, HBG2, ANXA4, and FHL1) which demonstrates the highest interaction between sub-anatomical region and sample site on protein intensity are shown in Fig. 2. No pattern was apparent, allowing the separate analysis of DEPs between sub-anatomical regions and sample sites.

Simple ANOVAs revealed no significant DEPs between sample sites. Sub-anatomical region comparison maintained the previously identified 374 DEPs. Maternal vs middle sub-anatomical region comparison revealed 128 upregulated, and 152 downregulated DEPs on the maternal side. Maternal vs fetal comparison revealed 115 upregulated, and 123 downregulated DEPs, respectively. Middle vs fetal sub-anatomical region comparison revealed 64 upregulated, and 41 downregulated DEPs in the middle sub-anatomical region (Table 2, Additional file 1: Figure 3).

**Table 2:** Summary of differentially expressed proteins (HGNC identifier) between maternal, middle, and fetal sub-anatomical regions

	Up DEP	Top 3 DEP	FC	Down DEP	Top 3 DEP	FC
Maternal vs middle sub-anatomical region	128	MYH11*** NUTF2* CFHR1***	1.48 1.01 0.80	152	PZP*** SCIN*** KDELC2***	− 1.41 − 1.15 − 0.79
Maternal vs fetal sub-anatomical region	115	MYH11*** COL14A1*** NUTF2**	1.24 1.16 1.00	123	KRT19*** SNRPD2** CAPS*	− 0.71 − 0.69 − 0.52
Middle vs fetal	64	PZP*** SCIN*** LTF***	2.29 0.80 0.70	41	DES*** SNRPD2** ERAP1***	− 0.74 − 0.66 − 0.46

Top three DEPs MYH11, NUTF2, CFHr1, COL14A1, NUTF2, PZP, SCIN, LFT based on highest fold change (FC). Adjusted p val < 0.05, up = upregulated, down = downregulated. *** = adjusted p val < 0.001, ** = adjusted p val < 0.01, * = adjusted p val < 0.05. p values adjusted for FDR

The top three up and downregulated DEP are detailed in Table 2. These proteins are associated with an array of molecular functions such as actin binding, protein binding, DNA binding, heparin binding, lipid binding and collagen binding.

### Functional analysis of placenta regions and sample site proteome

To further our understanding of functional differences between sub-anatomical regions based on associated DEP, functional analysis was conducted. DEP of maternal vs fetal, maternal vs middle, middle vs fetal sub-anatomical regions were directly compared within ClueGO regarding biological process gene ontology (GO), molecular function GO, and cellular component GO overrepresentation. As there was overlap between up and downregulated DEPs (Additional file 1: Figure S4), functional comparisons were separated to consider upregulated DEPs and downregulated DEPs [20].

**Fig. 3:**
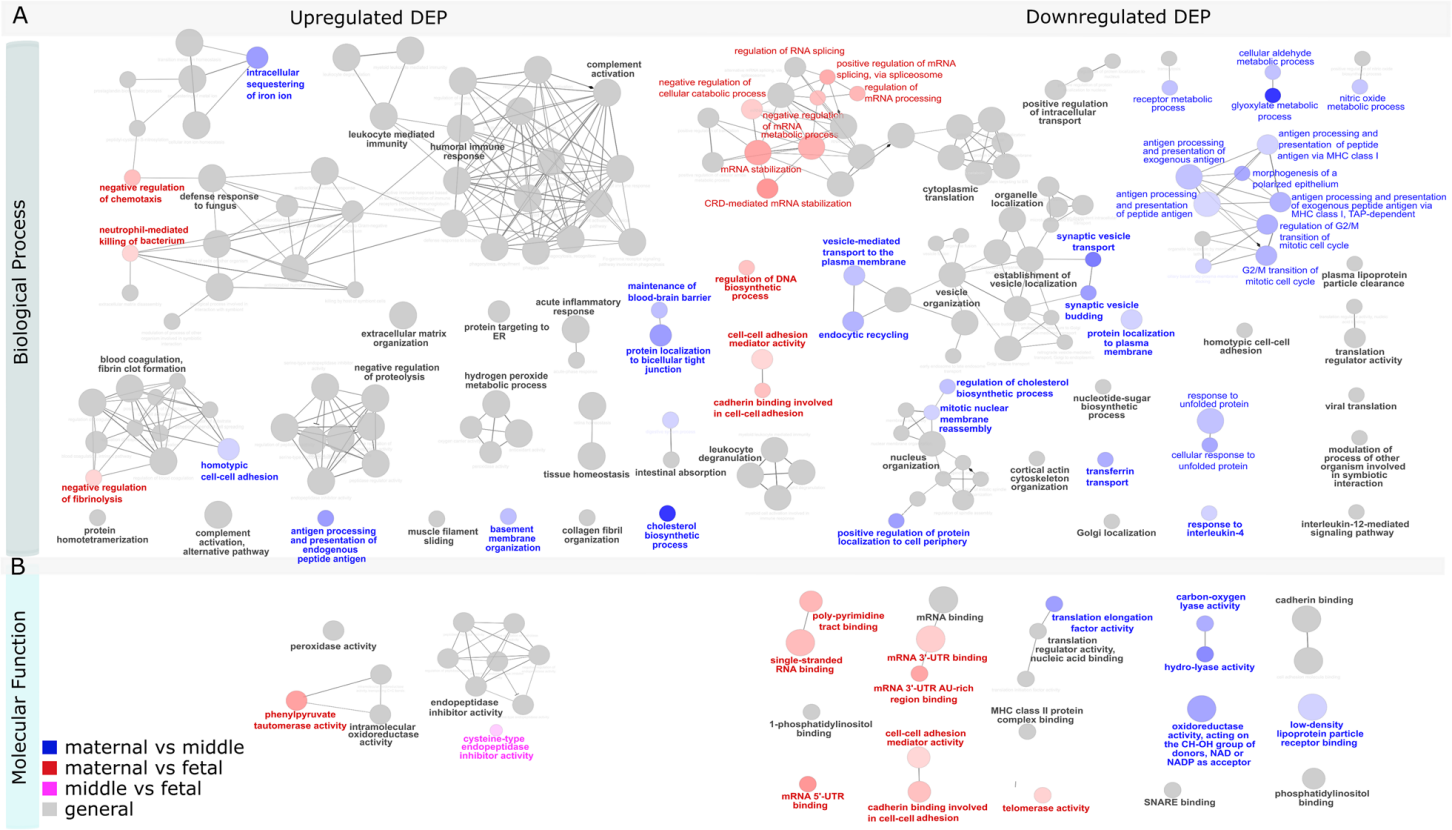
ClueGO functional analysis of differentially expressed proteins (DEP) between maternal, middle, and fetal sub-anatomical regions. Significantly (adj. p < 0.05) overrepresented biological process (**A**) and molecular function (**B**). GO terms can be grouped by similarity, in which case only the group GO term is legible. The size of nodes indicates the number of proteins encoding genes falling into each term. Similarity or hierarchic relationship between GO terms is indicated by edges

GO overrepresentation analysis indicated a number of biological processes and molecular functions to be enriched (Fig. 3). Processes and functions important in inflammation, humoral and stress responses were overrepresented in both up and down regulated proteins. Processes associated with transcription and translation, such as regulation of RNA, RNA catabolic processes, and regulation of DNA biosynthetic process were primarily overrepresented in downregulated DEPs in the maternal compared to the fetal sub-anatomical region. Processes associated with intracellular transport were primarily enriched in downregulated DEPs in the maternal compared to the middle sub-anatomical region (Table 3).

**Table 3:**
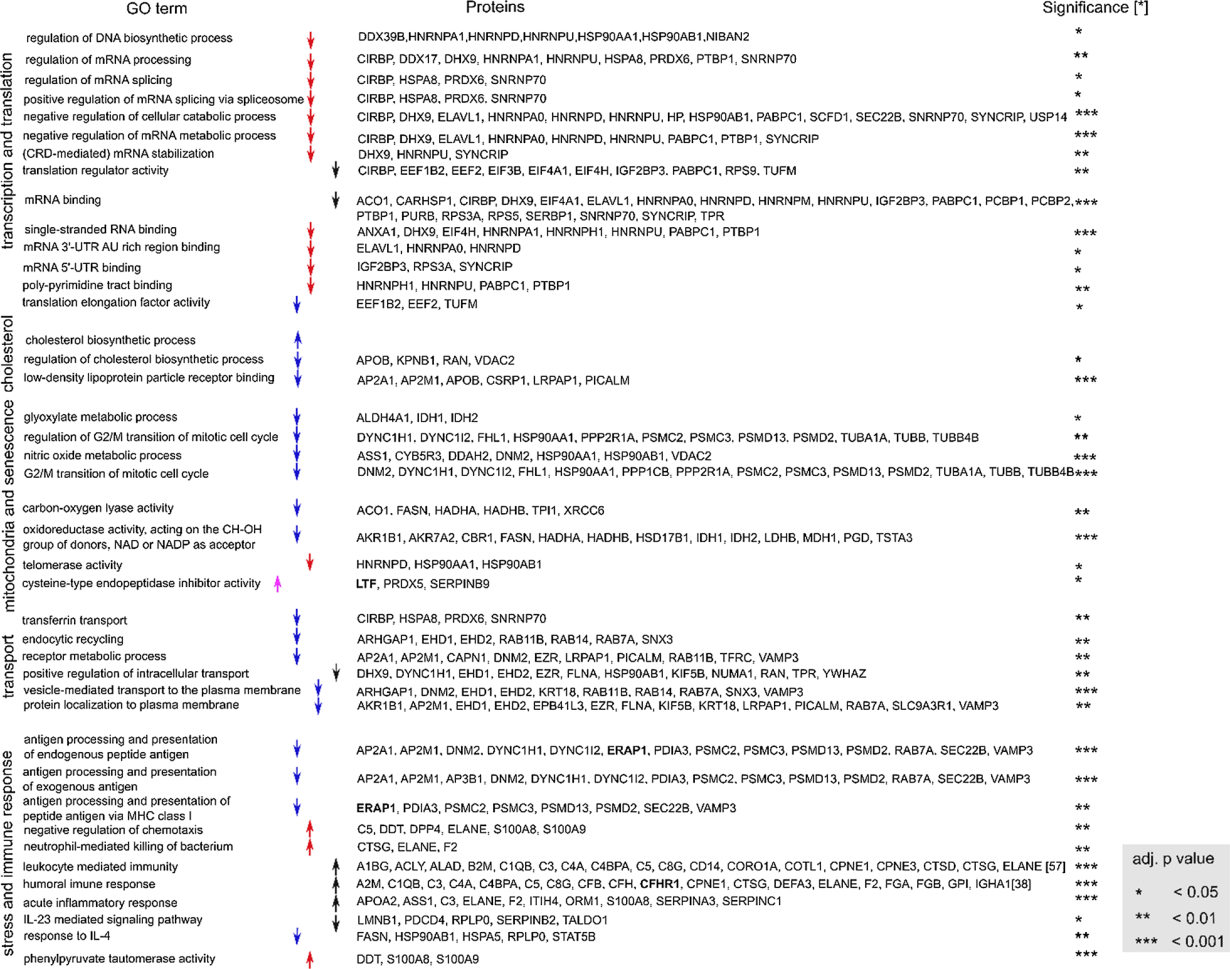
Summary table of GO terms overrepresented in DEPs between maternal and middle sub-anatomical region (blue), maternal and fetal sub-anatomical region (red), middle and fetal sub-anatomical region (pink), compared to each other

Mitochondria and senescence associated processes and functions such as, regulation of G2/M transition mitotic cell cycle, nitric oxide metabolism, oxidoreductase activity, and telomerase activity were overrepresented in downregulated DEPs between maternal and middle sub-anatomical regions. Proteins associated with glyoxylate metabolic processes were overrepresented in downregulated DEPs between maternal and middle or fetal sub-anatomical regions.

Terms that are overrepresented in general but not significant to one specific comparison group are visualized in grey. Upside arrows indicate upregulation, and downside arrows downregulation. Simplified results are summarized, including adj. p value (significance) indicator and associated proteins.

GO overrepresentation analysis revealed a myriad of differentially regulated processes and functions. In order to focus results further, and make them placenta specific, we used the NaviCenta, a placenta specific disease map. In contrast to traditional methods to characterize the function of deregulated genes or proteins the NaviCenta allows for consideration of DEPs including their respective fold change.

### Placenta specific functional analysis

Placenta specific phenotype prediction showed the proteins found in the maternal sub-anatomical region indicate increased endothelial cell (EC) migration, EC apoptosis, and EC proliferation in comparison to both middle and fetal sub-anatomical regions. The phenotypes contraction is higher in the maternal sub-anatomical region compared to both fetal and middle sub-anatomical regions, but lower in the middle sub-anatomical region compared to the fetal sub-anatomical region. Complement cascade and chemotaxis are lower in maternal and middle sub-anatomical regions compared to the fetal sub-anatomical region. Ferritinophagy is lower in the maternal than the middle sub-anatomical region, while mRNA splicing is higher in the maternal than the middle sub-anatomical region (Table 4).

**Table 4:** NaviCenta differential phenotype prediction based on DEP (Benjamini – Hochberg FDR correction) p-value including adj. Pval < 0.01 between maternal, middle, and fetal sub-anatomical regions and the proteins leading to each phenotype prediction

Phenotype	Mat-fet log2FC	pval	Gene Name	Mat-mid log2FC	pval		Mid-fet log2FC	pval	Gene Names
EC migration	0.28	5.94 e^−24^	PLG	0.18	3.76 e^−28^	PLG	0	1	
Inflammatory response	− 0.26	5.91 e^−21^	IGHG4	0	1		− 0.31	2.30 e^−35^	IGHG4
contraction	1	1.51 e^−13^	MYH11	1	5.41 e^−35^	MYH11, TAGLN	− 0.33	3.13 e^−22^	TAGLN
EC apoptosis	0.2	1.84 e^−11^	BLVRB	0.15	2.39 e^−13^	BLVRB	0	1	
EC proliferation	0.2	5.12 e^−11^	BLVRB	0.15	5.83 e^−13^	BLVRB	0	1	
Complement cascade	− 0.27	1.03 e^−08^	IGHG4	0	1		− 0.31	3.94 e^−19^	IGHG4
chemotaxis	− 0.27	3.91 e^−08^	IGHG4	0	1		− 0.31	5.30 e^−18^	IGHG4
Antigen clearance	0.41	0.003	IGHA1, IGLC3	− 0.05	0.070		0.63	2.22 e^−11^	IGHA1
Destruction of pathogens	0.15	0.008	MPO	0.07	0.048		0	1	
Ferritinophagy	0.2	0.453		− 0.14	0.001	FTL, FTH1	0	1	
mRNA splicing	0	1		0.12	0.003	ACIN1	0	1	

### Placental sub-anatomical regions present differing mitochondrial status

**Fig. 4:**
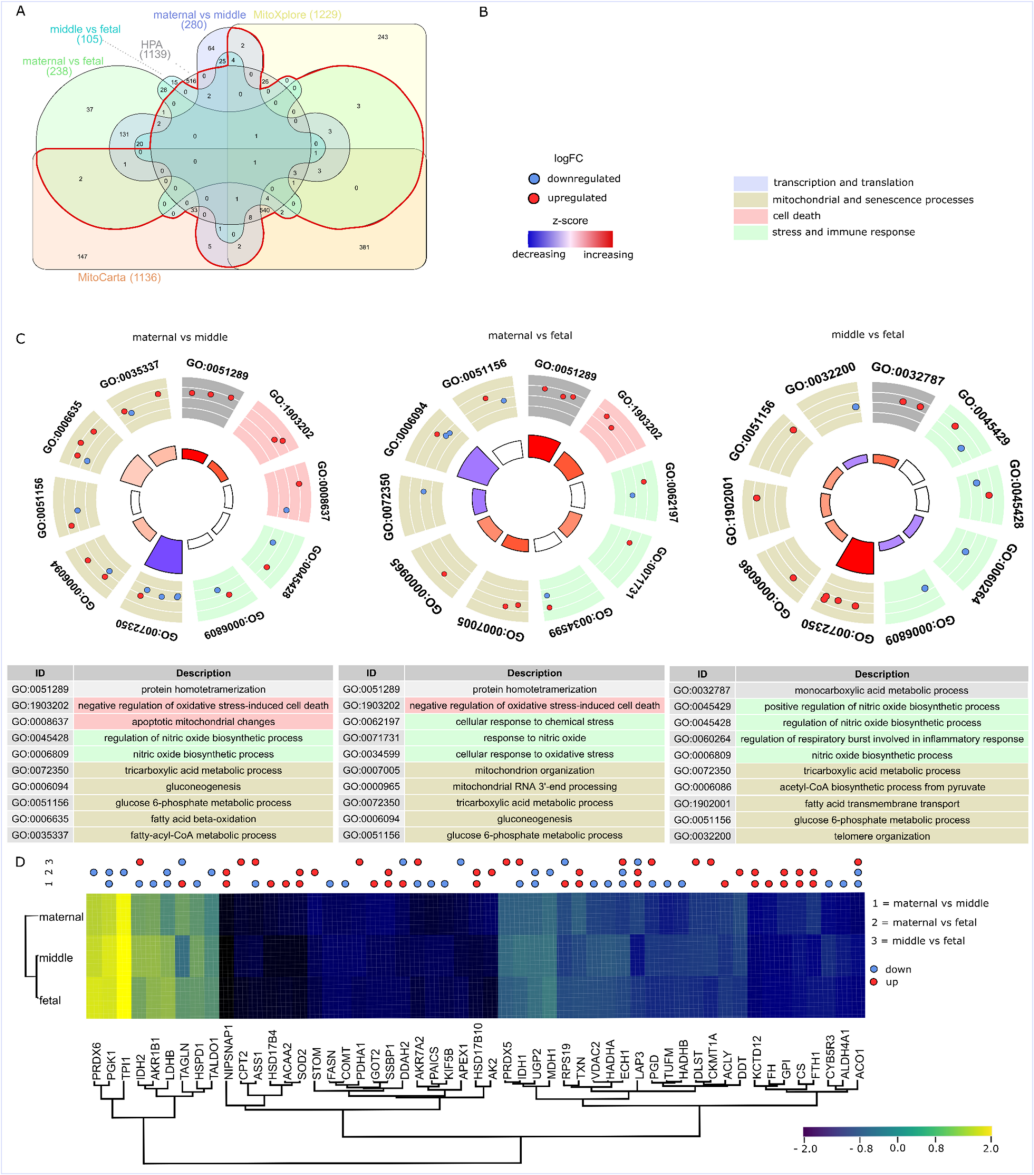
Mitochondria associated DEPs and their functional analysis. **A** Venn diagram of DEPs of maternal vs fetal, maternal vs middle, middle vs fetal, and proteins and genes associated with mitochondrial functions and processes from the databases MitoXplorer, human protein atlas (HPA)—mitochondria specific, and MitoCarta V3.0. The section of proteins used for further analysis is within the red border. **B** GO biological processes included and figure legend to **C**. GOCircle plots of the 10 most significantly overrepresented mitochondria associated with GO biological process terms, including DEPs falling into each term. Regulation direction is colour coded: upregulated (red), downregulated (blue). Size of the inner segments is indicative of the number of proteins falling into each term. The colour of each inner segment indicates the z-score—general up or downregulation of the GO term considering the number up- and downregulated proteins. **D** Clustered heatmap of average normalized LFQ intensities of mitochondria associated DEPs. Proteins are grouped into samples from maternal, middle, fetal sub-anatomical regions

The list of all identified proteins was filtered to only include proteins (HGNC IDs) included in the mitochondrial gene and proteome databases Human Protein Atlas mitochondrial sub-cellular location( www.proteinatlas.org), MitoXplorer human interactome [25], and human MitoCarta3.0 [26] (Fig. 4). HPA contains 1139 proteins associated with mitochondrial function, MitoCarta 3.0 1136, and MitoXplorer 1229, respectively (Fig. 4A). These databases showed overlapping content proteins both with each other and with the DEPs identified between sub-anatomical placenta regions. Ensuing functional analysis was conducted using the intersection of any mitochondrial database with placental proteins. Of all identified proteins 147 out of 1081 in the maternal sub-anatomical region, 144 out of 1086 in the middle sub-anatomical region, and 148 out of 1101 in the fetal sub-anatomical region are associated with mitochondrial function and processes. 53 DEPs out of the previous 374 DEPs are associated with mitochondrial function and processes.

The tricarboxylic acid cycle is indicated in all three conditional comparisons. Downregulated proteins are more evident on the maternal side compared with middle and fetal regions, and exclusively upregulated proteins comparing middle with fetal sub-anatomical region. Protein homotetramerization is indicated by upregulated proteins in the maternal region compared to both the fetal and the middle regions. Proteins associated with negative regulation of oxidative stress-induced cell death are upregulated in the maternal region compared with both middle and fetal regions. Summarizing the results, a general switch from glucose to lipid consumption appears to be initiated in the maternal and middle compared to the fetal sub-anatomical region. Terms indicating glycolysis, tricarboxylic acid cycle, and lipid consumption as an energy source are gluconeogenesis, glucose 6-phosphate metabolic process, fatty acid transmembrane transport, acetyl-CoA biosynthetic process from pyruvate, NADH regeneration, and fatty acid beta oxidation.

Comparing maternal and fetal sub-anatomical region, mitochondria associated terms such as mitochondrion organization and mitochondrial RNA 3’-end processing are upregulated in the maternal sub-anatomical region, while APEX1 associated with telomere organization is downregulated in the middle sub-anatomical region compared to the fetal sub-anatomical region.

### Placental sub-anatomical regions offer divergent senescence status

The difference in senescence state between the three sub-anatomical regions was analysed using three senescence specific databases. Human Ageing Genomic Resource (HAGR) CellAge Build 20, consisting of 307 genes (Avelar et al. 2020), Cell Senescence Gene Database (CSGene) consisting of 503 genes, and the National Centre for Biotechnology Information Search database (NCBI) specifically genes associated with senescence in homo sapiens consisting of 988 genes. Of all identified proteins 790 out of 1081 in the maternal sub-anatomical region, 794 out of 1086 in the middle sub-anatomical region, and 804 out of 1101 in the fetal sub-anatomical region were associated with senescence.

**Fig. 5:**
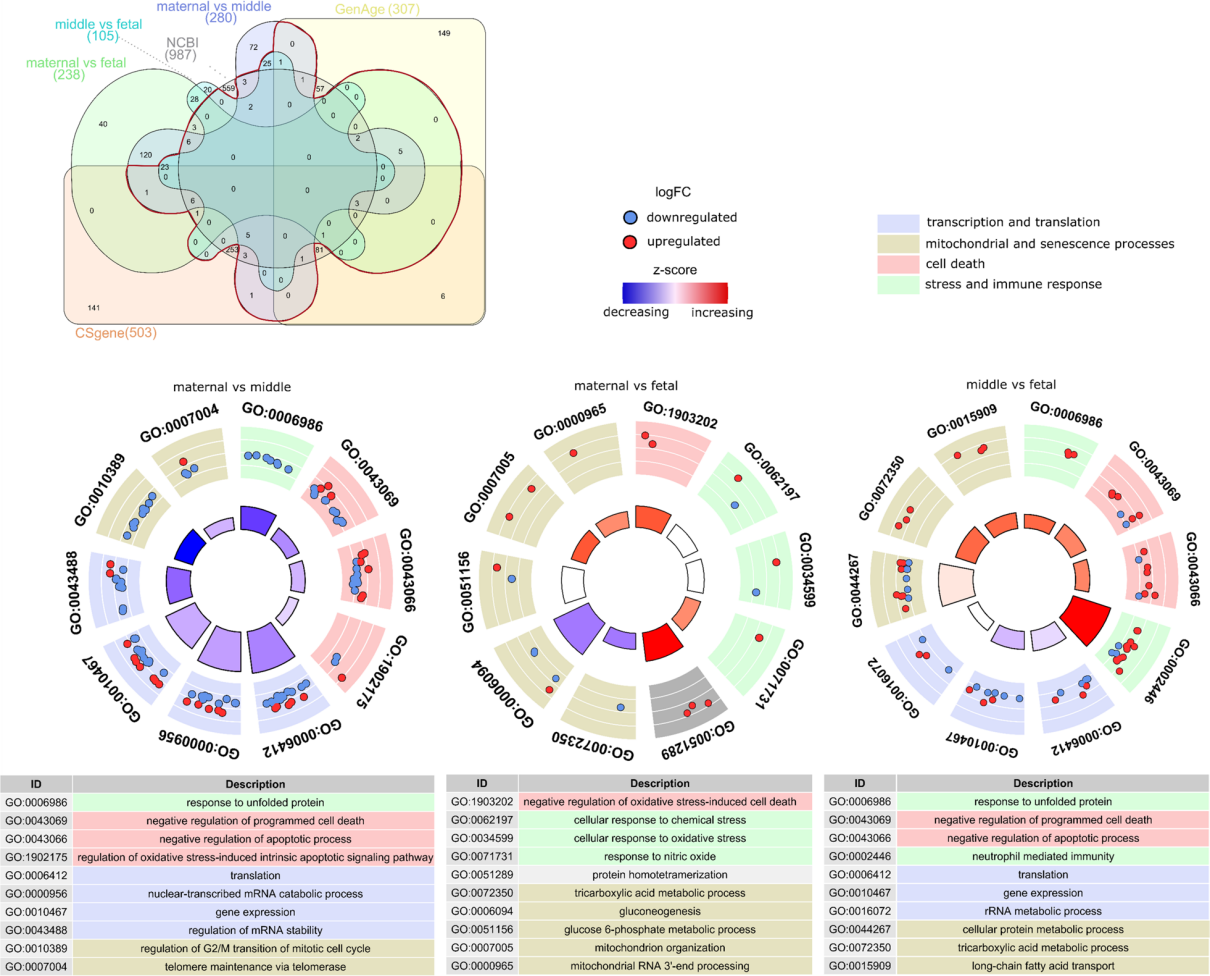
Senescence associated DEPs and their functional analysis. **A** Venn diagram of DEPs of maternal vs fetal, maternal vs middle, middle vs fetal, and proteins and genes associated with mitochondrial functions and processes from the databases CellAge, CSGene, and NCBI database results for the keyword senescence. The section of proteins used for further analysis is within the red border. **B** GOCircle plots of the 10 most significantly overrepresented GO biological process terms, including senescence associated DEPs falling into each term. Regulation direction is colour coded: upregulated (red), downregulated (blue). Size of the inner segments is indicative of the number of proteins falling into each term. The colour of each inner segment indicates the z-score—general up or downregulation of the GO term considering the number up- and downregulated proteins

In total, 290 of the previously 374 DEPs were associated with senescence (Fig. 5). Both transcription and translational processes (translation, nuclear-transcribed mRNA catabolic process, gene expression, regulation of mRNA stability) were differentially regulated between maternal and middle sub-anatomical region, with primarily down-regulated proteins on the maternal side, most of which were also downregulated in the middle compared to the fetal sub-anatomical region. Interestingly, proteins associated with negative regulation of, or regulation of cell death and apoptotic processes were primarily downregulated in the maternal compared to the middle sub-anatomical region and upregulated in the middle compared with the fetal sub-anatomical region.

Proteins associated with stress responses were downregulated in the maternal compared to the middle sub-anatomical region and primarily upregulated in the middle compared to the fetal sub-anatomical region. Other senescence associated proteins (regulation of G2/M transition of mitotic cell cycle, telomere maintenance) were primarily downregulated in the maternal compared to the middle sub-anatomical region.

## Discussion

The placenta is a highly complex organ and crucial for embryo development, however molecular interactions and their association with pregnancy complications remain poorly understood [32, 33].

In this study, for the first time, the proteomics structure of healthy placentae was investigated. Three placentae from healthy uncomplicated pregnancies were sampled at five sample sites, and further categorised into three sub-anatomical regions. We ensured reproducibility by ensuring that the 5 identical cross sections collected from the placenta which were further divided into three sub-anatomical regions, represented approximate sub-anatomical regions, i.e., maternal, mid and fetal through histopathological confirmation. Subsequent LFQ mass spectrometry analysis revealed distinct protein signatures of placental sub-anatomical regions. Resulting functional differences were characterized using functional analysis.

### Placental sub-anatomical regions differ in senescence states switch from glucose to fatty acids as a source for energy

Mitochondria and senescence associated processes and functions such as, regulation of G2/M transition mitotic cell cycle, nitric oxide metabolism, and telomere maintenance are overrepresented in downregulated DEPs between maternal and middle sub-anatomical regions, indicating increased cell-cycle arrest of cells in the maternal sub-anatomical region compared with the middle sub-anatomical region. Additionally, proteins associated with glyoxylate metabolic processes were overrepresented in upregulated DEPs between middle and fetal sub-anatomical regions, suggesting an age induced switch from glucose to fatty acids via the activation of glyoxylate cycle, as a source of energy [34]. A high number of DEPs were associated with senescence, some of which overlap with mitochondria associated proteins. Interestingly both maternal versus fetal and maternal versus middle sub-anatomical region comparison indicate a decrease in senescence associated protein levels. When inspected more closely these DEPs, such as DEAD-Box Helicase 17 (DDX17), and heterogeneous nuclear ribonucleoprotein A1 (HNRNPA1), between maternal and fetal sub-anatomical regions do not possess pro-senescent qualities but protective functions against senescence [35, 36].

Similarly, the DEPs trifunctional enzyme subunit alpha and beta (HADHA) and (HADHB), 3-hydroxyacyl-CoA dehydrogenase type-2 (HSD17B10), and L-lactate dehydrogenase B chain (LHDB) are associated with senescence, mitochondrial dysfunction, and the beta-oxidation pathway [37, 38]. Downregulation of HADHA, HADHB, and HSD17B10 have been shown to induce mitochondrial dysfunction and senescence [39]. LHDB is involved in the conversion of pyruvate to (S)-lactate; its down-regulation causes downregulation of mitochondrial function. Whereas the induction of LDHB expression causes enhanced mitochondrial function- including positive changes in mitochondrial respiratory subunits, mitochondrial membrane potential, ATP, NAD + /NADH ratio [40]. This indicates that the more senescent phenotype within the maternal sub-anatomical region is caused by the downregulation of mitochondrial proteins with anti-senescent properties.

The placentas collected are from term gestation (> 37 weeks of gestation), hence the ageing changes seen in the maternal sub-anatomical region is more physiologically rather than pathological. This hypothesis is also supplemented by lower ferritinophagy in the maternal sub-anatomical region. Ferritinophagy is a ferroptosis regulator and associated with iron-dependent regulated necrosis usually caused by massive lipid peroxidation-mediated membrane damage [41]. Moreover, we also report mitochondrial dysfunction in the form of glucose to lipid oxidation shift for energy production within the maternal sub-anatomical region compared to middle, or fetal sub-anatomical regions. This change can be indicative of change in trophoblastic microenvironment as a consequence of higher oxidative stress within this region [42].

### Mitochondrial activity is higher in the middle sub-anatomical region compared to maternal and fetal sub-anatomical regions

Mitochondrial-related DEPs were predominantly down-regulated on the maternal side compared with the middle sub-anatomical regions, and predominantly upregulated proteins comparing the middle with the fetal sub-anatomical region.

The upregulated proteins in the middle versus fetal sub-anatomical region, included IDH1, IDH2, ALDH4A1 and PDHA1. Isocitrate dehydrogenases (IDH) protein 1 found in the cytosol, while IDH2 is found in mitochondria and converts isocitrate to alpha-ketoglutarate (α-KG) in the TCA cycle and production of NADPH [43]. Upregulated protein expression of IDH1 and IDH2 levels in the middle region compared to maternal or fetal regions, suggests a higher production of NADPH and higher mitochondrial activity in this sub-anatomical region. In fact, studies have shown that IDH2 in pregnancy is inversely related to pregnancy conditions such as IUGR and high maternal BMI [44, 45]. Superoxide dismutase 2 (SOD2), which is upregulated in the middle sub-anatomical region compared to the fetal sub-anatomical region, is an enzymatic antioxidant that regulates oxidative stress, lack of antioxidant defence can lead to increased oxidative stress which in turn can damage cells, proteins, as well as DNA and accelerate aging. In pregnancy, this may lead to placental insufficiency linked to preeclampsia and IUGR [46, 47]. The increased expression of SOD2 along with IDH1, IDH2 indicate high energy homeostasis and metabolism, along with antioxidant defence in the middle sub-anatomical region [48, 49].

Apurinic/apyrimidinic endodeoxyribonuclease 1 or APEX-1 are multifunctional protein usually associated to DNA Damage Repair (DDR) functionality in cells experiencing oxidative insults [50]. Our results indicate that APEX-1 is downregulated in the middle sub-anatomical region compared to the fetal region. This might be indicative of higher oxidative stress experienced by this sub-anatomical region, causing telomere damage. The lack of indicators of oxidative stress within the fetal sub-anatomical region may be due to the presence of anti-senescent proteins, providing protection to the placental cells of this region. Moreover, upregulation of mitochondrial organization of genome and mitochondrial RNA 3’-end processing might be also indicative of mitochondrial stress response experience by the maternal sub-anatomical compared to fetal. However, the mechanism by which mitochondrial organization works is not completely understood [51].

### The effect of sub-anatomical region on protein intensity is not equal across sample sites

Independent analysis of unique proteins identified in placental sample sites revealed a myriad of proteins solely identified in one sample site E (Fig. 1) before normalisation. These were filtered out when adjusted for consideration of sub-anatomical regions. Additionally, strong interaction between the effect of sample site and sub-anatomical region on LFQ protein intensity was shown. This suggests that the effect between sub-anatomical region on LFQ proteins intensity is not the same for all sample sites. This may have been caused by individual variations and the placenta not being geometrically oval and stresses the importance of sampling several placenta sites from the same patient and recruiting an increased study number to compensate for the extremely high individual variability of placental shapes, size, and structure [16, 52]. Our sample collection protocol has been based on recommendation detailed by Burton et al., 2014 where we selected five different sites across the placenta [16]. As our results show, there is minute variations across the different sampling sites, with one site in particular showcasing unique proteins. We, therefore, concur with published recommendation for proteomic analysis by including at least five sites while sampling for both qualitative and quantitative validity [16]. We also identified proteomic differences between sub-anatomical regions, which we recommend considering in future studies. However, we did not include fetal and maternal membranes for sub-anatomical proteomic analysis. Through our results, we demonstrate a variable protein composition between sub-anatomical regions, with higher senescence associated protein and upregulated translation and transcription proteins in maternal and middle compared to fetal sub-anatomical region, hence we conclude separating these regions for a robust placental analysis.

### NaviCenta provides additional information to placental data analysis

Traditional functional analysis techniques such as GO terms are not tissue specific but consider all biological processes and molecular functions present within the human body. Even using the manually curated CellMarker database, the different placental cells could not be identified. Traditional functional analysis is unbiased. Although this is an advantage, in the context of placenta research, a more placenta focused approach is useful. The NaviCenta exclusively contains placenta tissue specific cells and phenotypes and enables network based in silico perturbations and the integration of log2FC, and p-value information. The NaviCenta indicated deregulated apoptosis, non-apoptotic cell death, and cell proliferation associated phenotypes of endothelial cells in particular [22]. These have been shown to be governed by mitochondrial function [53] and are associated with a more senescent state [54].

## Conclusion

This study provides a comprehensive proteomic assessment of separate sub-anatomical regions of the placenta reporting proteomic signature of healthy placentae, providing an insight into mitochondrial functioning and placental ageing. The proteome differences between placental sub-anatomical regions, referred to as maternal, middle, and fetal side, indicating cell senescence, mitochondrial dysfunction, and specifically activities of endothelial cells. Our results indicate a pronounced increase in senescence in both the maternal and middle sub-anatomical regions by downregulation of proteins protective against senescence. DEPs indicate additionally a senescence associated switch from ATP to fatty acids as a source of energy.

## Novelty and limitation

### Availability of official annotation for sub-anatomical regions

The sub-anatomical regions in this study are referred to as maternal, middle, and fetal sub-anatomical regions. In fact, the placenta is essentially a fetal organ and a selective barrier between maternal and fetal circulations [6]. The chosen nomenclature solely describes the location in reference to the foetus as described in Fig. 1 and was not intended to insinuate that there was transfer between maternal and fetal sub-anatomical region instead of the capillaries of the terminal villi.

### Individual variability of placental shape and structure

We observed that although there were no DEPs between the five placenta sample sites, some proteins showed increased interaction between sample site and sub-anatomical regions. This may have been caused by individual variations in placental morphology not being a geometrically perfect oval. This stresses the importance of extracting several placenta samples from the same patient and recruiting an increased number of participants to compensate for the extremely high individual variability of placental shapes, size, and structure [16, 52].

### Label free quantification

LC–MS techniques largely fall into two categories (i) relative quantification; and (ii) using a stable isotope-labelled standard to determine peptide abundance. Most research questions are addressed using relative quantification. Relative quantification can be further grouped into (i) labelled; and (ii) label-free. Samples can be labelled in vitro in the form of stable isotope labelling with amino acids in cell culture (SILAC) or modified using *i.e.,* tandem mass tag (TMT). These techniques diminish variability caused by sample preparation and make protein quantitation more exact [55, 56]. LFQ is a cost- and labour-effective alternative to both SILAC and TMT. The advantage here is that protein abundance alterations can be measured across samples independently of isotopic labelling. A disadvantage, however, is the acquisition time, as each sample must be run consecutively [57]. In this study, samples were minimally manipulated after extraction. No additional experimental conditions, but the proteomic differences between sub-anatomical regions were studied, making LFQ the preferred technique.

### NaviCenta—the placenta specific disease map

When it comes to disease or even tissue specific functional analysis and systemic data interpretation, computational and mathematical tools are not widely available, yet. The NaviCenta is a human and machine readable multiscale computational model. The NaviCenta consists of information regarding processes and cell interactions within the placenta, and of relevance to healthy placentation, including molecular interactions between proteins, RNA, and DNA, enriched with regulatory factors, such as transcription factors, miRNA, and lncRNA, which can be used for network-based analyses [21]. In this study, the NaviCenta was used to perform placenta specific phenotype prediction, also considering protein fold change, which the general functional analysis was integrated into, enabling a more meaningful interpretation of results.

## Supplementary Information

**Additional file 1.** Supplementary figures and functional analysis of DEPs between placental sub-anatomical regions.

**Additional file 2.** Table of DEPs between sub-anatomical regions.

## Data Availability

**Functional analysis of proteins identified in placental sub-anatomical regions: **Original EnrichR results are available under. Maternal: https://maayanlab.cloud/Enrichr/enrich?dataset=9c376a64adf43c8c8c1782694549483e, accessed 29 December 2021. Middle: https://maayanlab.cloud/Enrichr/enrich?dataset=27fb12bd286394d15787f76f1e9dc3d4, accessed 29 December 2021. Fetal: https://maayanlab.cloud/Enrichr/enrich?dataset=60a1314c656e72744ea87b28d82c475a, accessed 30 December 2021. The raw mass spectrometry data is privately available on Fairdom hub upon request. **Mitochondrial proteins and genes: **Human protein atlas information specific to the mitochondrial subcellular location was downloaded from https://www.proteinatlas.org/search/subcell_location:Mitochondria. The mitochondrial interactome from MitoXplorer was downloaded from http://mitoxplorer.ibdm.univ-mrs.fr/interactome.php. Human MitoCarta 3.0 was downloaded from https://www.broadinstitute.org/mitocarta/mitocarta30-inventory-mammalian-mitochondrial-proteins-and-pathways. **NaviCenta:** The placenta specific disease map is available under https://www.sbi.uni-rostock.de/minerva/index.xhtml?id=NaviCenta. Plugins used for phenotype prediction are available upon request. **Mitochondrial and Senescence Data:** Mat-fet: https://maayanlab.cloud/Enrichr/enrich?dataset=23a1f5d1e2214762a57c6e9d41a4b006. Mito mat-mid: https://maayanlab.cloud/Enrichr/enrich?dataset=a0339b7b961cadf671ad33f94a050c5c. Mito mid-fet: https://maayanlab.cloud/Enrichr/enrich?dataset=b6eaac4d63fa78e94acac4c9cf4506da. **Enrichr bp:** Mat-fet: https://maayanlab.cloud/Enrichr/enrich?dataset=d2f9dc83798e33f0159517ba9df04fba. mat-mid: https://maayanlab.cloud/Enrichr/enrich?dataset=ac55a8568727078143b9bace07f1b013. mid-fet: https://maayanlab.cloud/Enrichr/enrich?dataset=6b734e65e15f5aeb003e23e4c574ea50.
