# Supplementary material for: A proteomic profile of the healthy human placenta
Source: Clin Proteomics. 2023 Jan 2;20:1. doi: 10.1186/s12014-022-09388-4 (PMC9808999; doi:10.1186/s12014-022-09388-4)
Supplement: Supplementary file 1 — Additional file 1. Supplementary figures and functional analysis of DEPs between placental sub-anatomical regions. [file 12014_2022_9388_MOESM1_ESM.docx]

# Additional


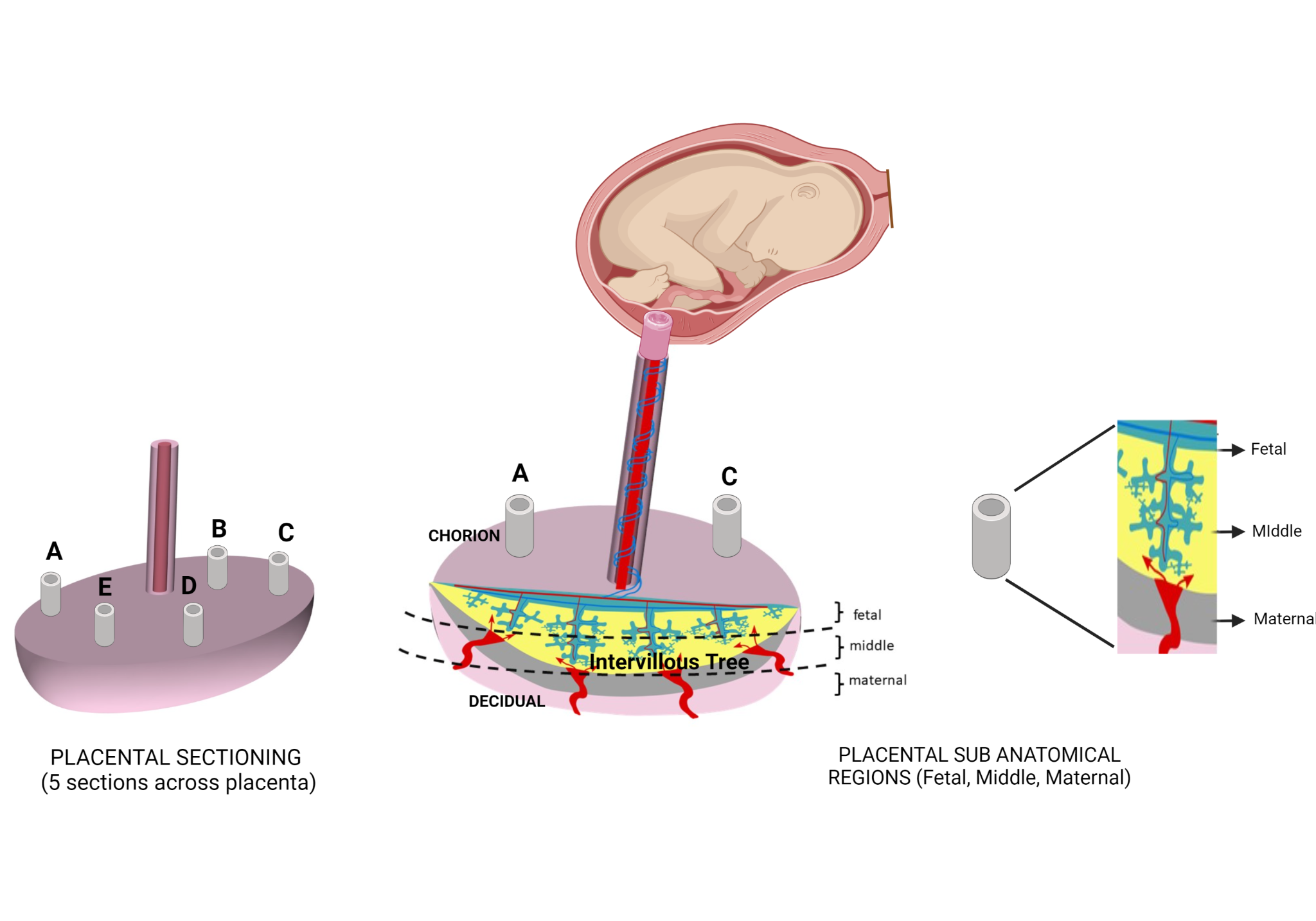


**
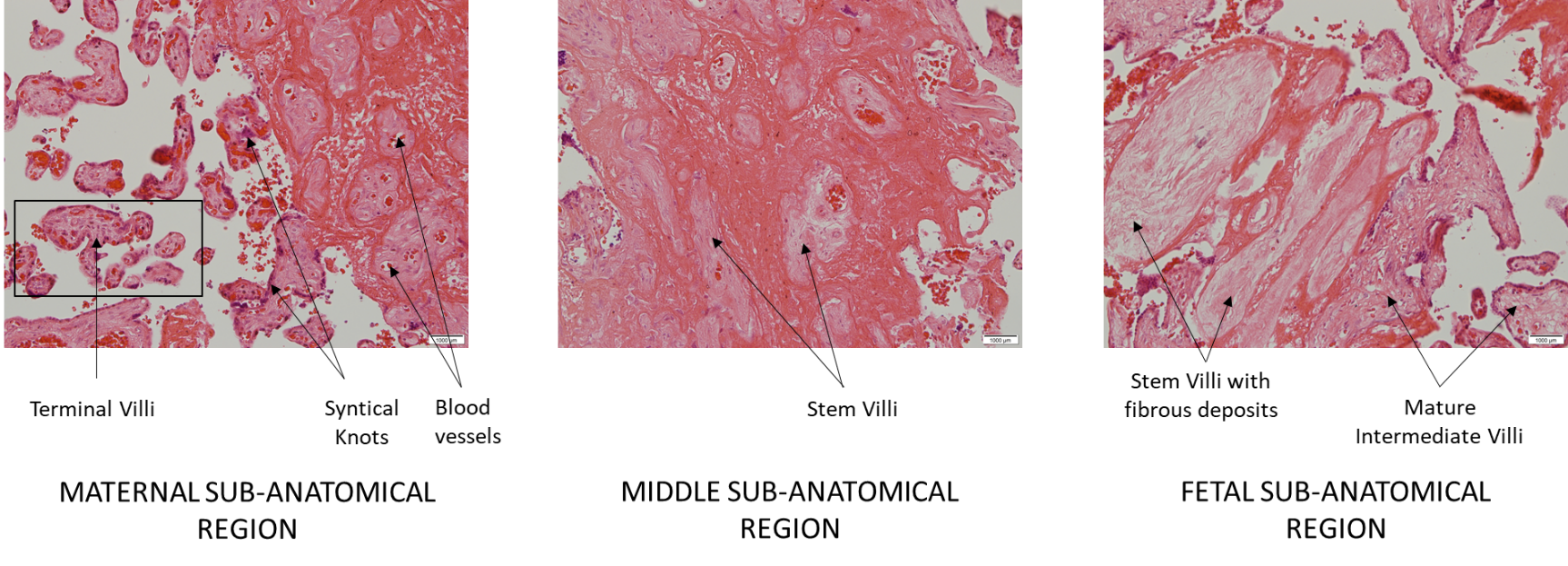
Histopathological sectioning:**

Additional Figure S1: Histopathological classification of sub-anatomical region sectioning. The maternal region shows evidence of developed terminal villi and arterial vessels, indicating maternal side of the placenta. The fetal sub-anatomical region with mature intermediate villi which provide fetal vascularization for feto-maternal exchange, along with a high fibrous deposition, suggesting it is the fetal side of the placenta. The middle region shows increased evidence of stem villi. Stem Villi are made up of trunks and branches of the villous tree and anchoring villi, depending on caliber and position they assume. At term, they are present mostly in subchorionic areas of the placenta, providing mechanical support to the organ [52].

**Uniquely Identified proteins in placental sub-anatomical regions:** Please see supplementary material 2 or a detailed version of this list can be found on https://fairdomhub.org/data_files/6226.

**Functional analysis of identified proteins in sub-anatomical regions**

To get a general overview of the proteins identified within samples, EnrichR Cell Marker Database [25] was used to predict cell types based on proteome profiles. Considering cell type prediction exclusively, there were only minor differences between sub-anatomical regions (Supplemental Figure 3). Although placental tissues were not specifically implicated, most enriched cell types indicate fetal tissues, such as monocytes from fetal kidney, natural killer T (NKT) cells from fetal kidney, mitotic fetal germ


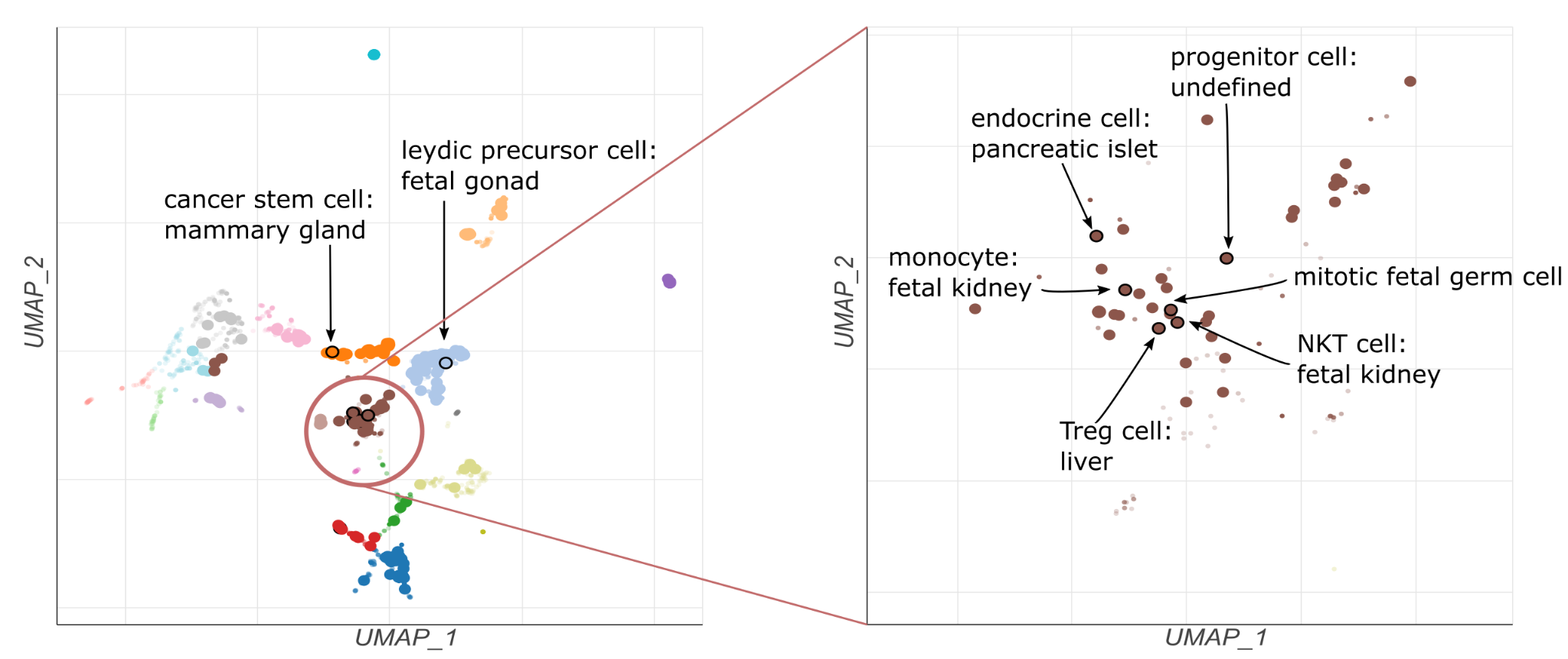


Supplemental Figure 2: Exemplary scatter plot of cell type prediction based on identified proteins within placenta samples using EnrichR CellMarker Augmented 2021. Similar gene sets are clustered together and in the same color. Significant enrichment is visualized as a black border and labelled. The cluster with a high number of significantly enriched cell types was further enlarged for legibility.

**EnrichR results of unique proteins in sub-anatomical regions**

Fetal BP: regulation of IRE1-mediated unfolded protein response (GO:1903894)

Middle BP

| \| signal transduction in response to DNA damage (GO:0042770) \| \| --- \| \| mRNA 3'-splice site recognition (GO:0000389) \| \| peptidyl-lysine hydroxylation (GO:0017185) \| \| entry of bacterium into host cell (GO:0035635) \| \| RNA splicing, via transesterification reactions with bulged adenosine as nucleophile (GO:0000377) \| \| mRNA splicing, via spliceosome (GO:0000398) \| \| cellular response to interleukin-15 (GO:0071350) \| \| interleukin-15-mediated signaling pathway (GO:0035723) \| \| entry into host (GO:0044409) \| \| mRNA processing (GO:0006397) \| \| neurotrophin TRK receptor signaling pathway (GO:0048011) \| | \| protein hydroxylation (GO:0018126) \| \| --- \| \| heme catabolic process (GO:0042167) \| \| porphyrin-containing compound catabolic process (GO:0006787) \| \| hydrogen peroxide catabolic process (GO:0042744) \| \| neurotrophin signaling pathway (GO:0038179) \| \| RNA splicing, via transesterification reactions (GO:0000375) \| \| hydrogen peroxide metabolic process (GO:0042743) \| \| mRNA splice site selection (GO:0006376) \| \| positive regulation of small GTPase mediated signal transduction (GO:0051057) \| \| ERBB2 signaling pathway (GO:0038128) \| \| DNA damage checkpoint signaling (GO:0000077) \| |
| --- | --- | --- | --- | --- | --- | --- | --- | --- | --- | --- | --- | --- | --- | --- | --- | --- | --- | --- | --- | --- | --- | --- | --- |

Maternal BP

| \| \| negative regulation of transforming growth factor beta receptor signaling pathway \| \| --- \| \| regulation of transforming growth factor beta receptor signaling pathway \| \| negative regulation of transmembrane receptor protein serine/threonine kinase signaling pathway \| \| membrane fusion \| \| positive regulation of intrinsic apoptotic signaling pathway \| \| regulation of intrinsic apoptotic signaling pathway \| \| positive regulation of protein binding \| \| positive regulation of apoptotic signaling pathway \| \| cholesterol homeostasis \| \| sterol homeostasis \| \| positive regulation of protein modification by small protein conjugation or removal \| \| positive regulation of protein ubiquitination \| \| positive regulation of binding \| \| negative regulation of MAPK cascade \| \| regulation of protein ubiquitination \| \| calcium ion transport \| \| regulation of protein binding \| \| negative regulation of calcium ion transport into cytosol \| \| regulation of inward rectifier potassium channel activity \| \| negative regulation of metallopeptidase activity \| \| caveola assembly \| \| caveolin-mediated endocytosis \| \| negative regulation of pinocytosis \| \| protein localization to membrane raft \| \| intracellular distribution of mitochondria \| \| positive regulation of extrinsic apoptotic signaling pathway via death domain receptors \| \| positive regulation of toll-like receptor 3 signaling pathway \| \| positive regulation of gap junction assembly \| \| plasma membrane raft assembly \| \| SMAD protein complex assembly \| \| angiotensin-activated signaling pathway \| \| receptor-mediated endocytosis of virus by host cell \| \| gas homeostasis \| \| positive regulation of cytoplasmic translation \| \| membrane fission \| \| positive regulation of ER-associated ubiquitin-dependent protein catabolic process \| \| definitive hemopoiesis \| \| heart trabecula formation \| \| negative regulation of membrane protein ectodomain proteolysis \| \| protein localization to basolateral plasma membrane \| \| regulation of entry of bacterium into host cell \| \| regulation of toll-like receptor 3 signaling pathway \| \| regulation of cardiac muscle cell action potential involved in regulation of contraction \| \| dynamin family protein polymerization involved in membrane fission \| \| dynamin family protein polymerization involved in mitochondrial fission \| \| negative regulation of canonical Wnt signaling pathway \| \| positive regulation of intracellular signal transduction \| \| regulation of gap junction assembly \| \| regulation of cell adhesion molecule production \| \| mitochondrial fragmentation involved in apoptotic process \| \| peroxisome fission \| \| negative regulation of nitric oxide biosynthetic process \| \| negative regulation of nitric oxide metabolic process \| \| regulation of pinocytosis \| \| regulation of ER-associated ubiquitin-dependent protein catabolic process \| \| positive regulation of receptor recycling \| \| negative regulation of protein autophosphorylation \| \| negative regulation of Wnt signaling pathway \| \| cellular response to increased oxygen levels \| \| negative regulation of calcium ion transmembrane transport \| \| negative regulation of voltage-gated potassium channel activity \| \| mitochondrion morphogenesis \| \| regulation of coagulation \| \| PERK-mediated unfolded protein response \| \| embryonic eye morphogenesis \| \| negative regulation of potassium ion transmembrane transport \| \| negative regulation of receptor signaling pathway via STAT \| \| negative regulation of ryanodine-sensitive calcium-release channel activity \| \| regulation of amyloid precursor protein catabolic process \| \| regulation of ventricular cardiac muscle cell action potential \| \| regulation of cell communication by electrical coupling involved in cardiac conduction \| \| mitochondrion distribution \| \| \| --- \| --- \| --- \| --- \| --- \| --- \| --- \| --- \| --- \| --- \| --- \| --- \| --- \| --- \| --- \| --- \| --- \| --- \| --- \| --- \| --- \| --- \| --- \| --- \| --- \| --- \| --- \| --- \| --- \| --- \| --- \| --- \| --- \| --- \| --- \| --- \| --- \| --- \| --- \| --- \| --- \| --- \| --- \| --- \| --- \| --- \| --- \| --- \| --- \| --- \| --- \| --- \| --- \| --- \| --- \| --- \| --- \| --- \| --- \| --- \| --- \| --- \| --- \| --- \| --- \| --- \| --- \| --- \| --- \| --- \| --- \| --- \| --- \| | \| negative regulation of release of sequestered calcium ion into cytosol \| \| --- \| \| regulation of membrane repolarization \| \| positive regulation of ERAD pathway \| \| regulation of cell communication by electrical coupling \| \| cell part morphogenesis \| \| negative regulation of potassium ion transport \| \| cellular response to exogenous dsRNA \| \| regulation of cytoplasmic translation \| \| integrated stress response signaling \| \| negative regulation of anoikis \| \| regulation of activin receptor signaling pathway \| \| negative regulation of epithelial cell differentiation \| \| positive regulation of vasoconstriction \| \| necroptotic process \| \| negative regulation of receptor signaling pathway via JAK-STAT \| \| regulation of receptor recycling \| \| regulation of fatty acid metabolic process \| \| epithelial structure maintenance \| \| negative regulation of cation transmembrane transport \| \| maintenance of gastrointestinal epithelium \| \| release of cytochrome c from mitochondria \| \| mitochondrial fission \| \| programmed necrotic cell death \| \| cellular response to dsRNA \| \| regulation of canonical Wnt signaling pathway \| \| positive regulation of neutrophil chemotaxis \| \| regulation of insulin-like growth factor receptor signaling pathway \| \| regulation of autophagy of mitochondrion \| \| heart trabecula morphogenesis \| \| positive regulation of mitochondrial fission \| \| regulation of epithelial cell differentiation \| \| regulation of smooth muscle contraction \| \| eye morphogenesis \| \| modulation by symbiont of entry into host \| \| protein-containing complex assembly \| \| sterol transport \| \| regulation of anoikis \| \| beta-catenin destruction complex disassembly \| \| response to progesterone \| \| negative regulation of peptidyl-serine phosphorylation \| \| positive regulation of neutrophil migration \| \| regulation of membrane protein ectodomain proteolysis \| \| positive regulation of granulocyte chemotaxis \| \| negative regulation of protein dephosphorylation \| \| positive regulation of cholesterol efflux \| \| regulation of peptidase activity \| \| negative regulation of phosphoprotein phosphatase activity \| \| regulation of ruffle assembly \| \| mammary gland development \| \| regulation of monooxygenase activity \| \| negative regulation of protein tyrosine kinase activity \| \| regulation of ryanodine-sensitive calcium-release channel activity \| \| negative regulation of endocytosis \| \| positive regulation of release of cytochrome c from mitochondria \| \| regulation of cardiac muscle cell action potential \| \| regulation of mitochondrial fission \| \| divalent inorganic cation homeostasis \| \| ventricular cardiac muscle tissue development \| \| regulation of neutrophil chemotaxis \| \| peroxisome organization \| \| organelle fission \| \| extracellular matrix organization \| \| protein peptidyl-prolyl isomerization \| \| negative regulation of cation channel activity \| \| regulation of blood coagulation \| \| regulation of cardiac muscle cell contraction \| \| positive regulation of toll-like receptor signaling pathway \| \| negative regulation of calcium ion transmembrane transporter activity \| \| regulation of muscle contraction \| \| response to ketone \| \| negative regulation of phosphatase activity \| |
| --- | --- | --- | --- | --- | --- | --- | --- | --- | --- | --- | --- | --- | --- | --- | --- | --- | --- | --- | --- | --- | --- | --- | --- | --- | --- | --- | --- | --- | --- | --- | --- | --- | --- | --- | --- | --- | --- | --- | --- | --- | --- | --- | --- | --- | --- | --- | --- | --- | --- | --- | --- | --- | --- | --- | --- | --- | --- | --- | --- | --- | --- | --- | --- | --- | --- | --- | --- | --- | --- | --- | --- | --- | --- | --- | --- | --- | --- | --- | --- | --- | --- | --- | --- | --- | --- | --- | --- | --- | --- | --- | --- | --- | --- | --- | --- | --- | --- | --- | --- | --- | --- | --- | --- | --- | --- | --- | --- | --- | --- | --- | --- | --- | --- | --- | --- | --- | --- | --- | --- | --- | --- | --- | --- | --- | --- | --- | --- | --- | --- | --- | --- | --- | --- | --- | --- | --- | --- | --- | --- | --- | --- | --- | --- | --- | --- |

**Functional analysis of unique proteins found in sample site 5**

The original LFQ intensities were imported into Perseus. Per sample site 9 individual samples were available. Contaminants were removed from further analysis. Proteins with less than 5 valid values (samples) were removed from further analysis.

Sample site yellow contained HTRA1 as a unique protein, whereas sample sit brown contained 520 unique proteins. ClueGO functional analysis for biological processes was performed on the unique proteins identified within sample site brown.

After rigorous filtering to assure reproducibility, including filtering for valid values in at least 100% of biological replicates, 70% valid values of samples per sample site, and 70% valid values of samples per sub-anatomical region, these proteomic differences did not persist.

| IGLV4-69  IGLV3-9  IGKV2-24  CPB2  PGM3  DNAJA2  TTR  UBXN1  SPCS2  OSTC  PSMD1  TRAPPC3  GMPR2  PDLIM3  C11orf31  LPP  BOLA2B  SNX12  COL6A1  SEPTIN8  RAB18  NDUFA5  GPX3  AGRN  C21orf33  DOCK1  TTN  SYNE2  IGKV3D-11  AQPEP  IARS  MUC15  FKBP8  SULT1A3  PRPS1  TRPV1  ACAA2  IGHV1-3  DLG1  ARSA  PIK3C2A  LAMTOR5  SEMA3B  ENOPH1  IGKV3D-20  IGHV1-18  IGKV1-6  EWSR1  CDKN1C  GBA  IGHA2  HLA-G  AIM1  LIMS1  IGHV3-23  PROCR  GNL1  PLEKHA6  RBMS1  HSD17B12  HMBS  SCARB2  HSPB6  PHGDH  EPB41 | MPST  STX12  GUK1  MFAP5  LMCD1  ABHD14B  ARMC3  PPM1F  NAP1L1  CNRIP1  VAMP8  AGFG1  CUTA  DCUN1D1  PPP1R7  EEF1E1  ARF5  PSMD6  COX17  PRR14L  SSR3  APOD  CD163  TFG  ATP5J2-PTCD1  GPD1L  FARP1  PPIH  DFNA5  ACP1  ABCE1  SAR1A  TMEM33  COPS4  LMAN2  EIF4E  JCHAIN  GCLC  ATOX1  PFDN1  MAT2B  NDRG1  CTHRC1  G6PD  AP1M1  COL6A3  ADD1  AIM1L  CD36  SLCO2A1  DKFZp686E0752  SEC24D  TNC  ACADS  TBL2  COPS8  LANCL1  CHID1  PRKCDBP  BCLAF1  PRMT1  NEDD8  ARRB1  DERA  PRCP | RAB8B  RNPS1  HEXA  MACF1  GSPT1  UQCRC2  COX5A  GCA  SRPRB  PSMD9  DDX5  SCO1  CDK5RAP3  RPL36  SON  BUB3  GALK1  KATNAL2  PSMD8  FARSA  DAZAP1  CDC37  EIF1  SF3A2  EIF3G  HDHD2  KDSR  COPE  ETHE1  SNRPA  AP2S1  GGCT  EPS15L1  PAFAH1B3  AIP  QSOX1  DNM1L  VWA5A  PPP6C  PTGES  SCAMP3  TAX1BP3  SCAMP2  SURF4  SPTLC1  EIF3D  YKT6  ARPC5  NARS  NUDT21  GMFG  SMARCA5  REV3L  MAFK  HIST1H2BK  EDF1  PSIP1  BANF1  SF3B1  RP2  EIF3J  DNAJC8  ATP5H  SRP72  VAPB | NQO2  H2AFX  PECAM1  HIST1H1D  EPB42  ATP2A2  HSPA6  LBP  ATP5J  ORM2  ATP2B1  EIF2S2  RAB5A  OSBP  CYP7A1  MYL9  MCM3  TARS  PON1  MAPK3  PSM8  MAPK1  GRN  CRABP2  LMOD1  ATP5D  NMT1  DNAJA1  CDA  GBP1  HSPA1L  TIMP3  ARL3  HPCA  GARS  ACTR1B  ECI1  MCAM  STT3A  PIP4K2A  NES  SEPHS1  PSMD7  PRELP  CNN1  SMS  BLVRA  SLC25A1  DAPK1  CTSC  AIF1  ADK  ITGA1  SEC61B  RAB5B  RAB10  VBP1  UFM1  RRAS2  LSM3  SNRPD2  RPL39P5  FKBP1A  GRB2  GNAI1 | PKIB  CHCHD2  LHPP  KIF24  NEK6  FAM46A  HBM  VASN  TWF2  HIBCH  C16orf46  DNMBP  HSDL2  NCCRP1  MOB1A  TMED4  KTN1  NIT1  CARKD  SLC35F6  SLC43A2  NHLRC2  COLGALT1  RDH13  SCCPDH  APOA1BP  NUP43  TSTD1  UBA3  NEK9  EPS8L1  ZNF721  TMEM40  PALLD  FAM3C  CCT6B  GCN1L1  NUP205  ARPC1A  COPS5  KCNB2  TNPO1  USP9X  EFHD2  TONSL  PAWR  PRRC1  PVRL4  S100A13  CHP1  SH3GL1  ARPC5L  ESYT1  LRRC1  C9orf142  PDCD10  TUBB2B  TMEM109  HINT2  NIF3L1  RAB1B  WDR13  EHD4  SLK  ALG2 | LIMA1  C12orf10  METAP2  FKBP11  COPZ1  PPP1CC  SDSL  UBAP2L  TES  GAPVD1  ATG3  ESAM  NIT2  API5  LIN7C  SHMT2  FBLN5  TMPO  NAPRT  ERC1  SMC1A  CD44  NCSTN  NEB  AHCYL2  TKFC  UBQLN1  EIF3F  ABCF1  MARS FUCA1  HRG  IGKV2-30  IGKV4-1  PYGL  CTSL  MGP  COL4A2  SERPINF2  HBQ1  SNRPA1  COX6C  C4B  UQCRFS1  POTEI  COX5B  PDHB  PSG2  PCNA  HARS  CKB  PEPD  COX4I1  C6  PRKAR2A  FDPS  COX6B1  JUP  CD46  NME1  VPS36  THRAP3  PRPF40A  DEK  SUCLG1 | SSRP1  NSUN2  SF3A3  FLII  SPP2  DNAJC3  EIF4EBP2  TUBB2A  FHL2  EIF4A2  RCN2  GNA13  RAB39A  EFTUD2  RAB3GAP1  SAFB  SF3A1  SLC9A3R2  PSMD5  HIST2H2BE  UPP1  RABL6  TUBB8  FAM98B  PHLDA2  ACTBL2  HSP90AB2P  HIST1H2BH  SNX5  GSN  THUMPD1  FAM120A  THYN1  DNAH1  DNAJB11  SRP68  PCYOX1  PFDN2  SH3BGRL2  DCTN4  HSPB8  AGO2  RAB21  SMC3  FNDC3A  AP3M1  NOP58  LSM2  STRAP  USP15  PPME1  SUPT16H  SNX9  CPQ  EPN1  LAMC3  ZFP37  SACM1L  ACP2  IGLC7  UNC80  STAT1  USP7  SSC5D  RPRD1B | CD47  TNS1  CSNK2A1  SUCLA2  SNX27  SEC23B  AP1S1  ADAR  GPNMB  COL4A1  STIM1  TOLLIP  CCS  SF3B2  PFDN4  LSM8  RAB35  GNS  XRCC1  TINAGL1  LYPLA2  SDPR  CYB5A  MT-CO2  HPRT1  F9  MT-ATP6  HBE1  COL1A1  CSNK2B  TUBA1B  MRPS36  FBLN2  PSG6  PLOD1  CAV1  SRSF11  KHDRBS1  FN3KRP  ACOT13  DIABLO  AASDHPPT  LANCL2  IARS2  FAM49B  PARVA  ARL8B  TPP2  SART3  LRP8  SRSF6  NIPBL  ABHD12  MLLT4  EIF3L  BAX  HCFC1  DPRX  NUDT5  HN1L  EIF1AX  CLIC2  PSMD4  C1orf123  C1QA |
| --- | --- | --- | --- | --- | --- | --- | --- |

Supplemental Table 1: Proteins identified in sample site brown.


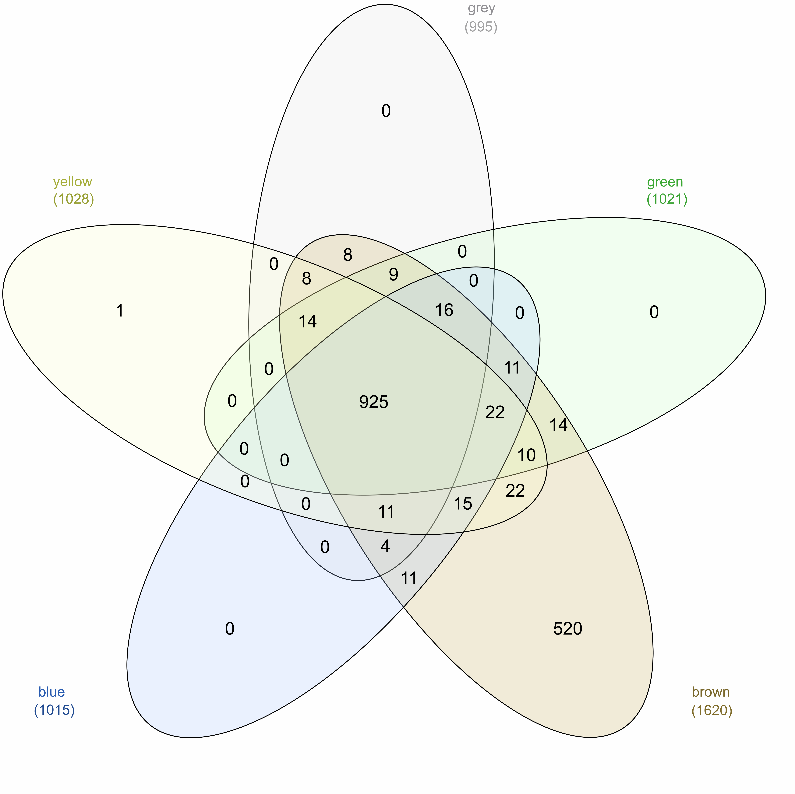


Supplemental Figure 3: Venn diagram of identified proteins in sample sites. Numbers in brackets signify the total number of identified proteins within respective sampling sites.


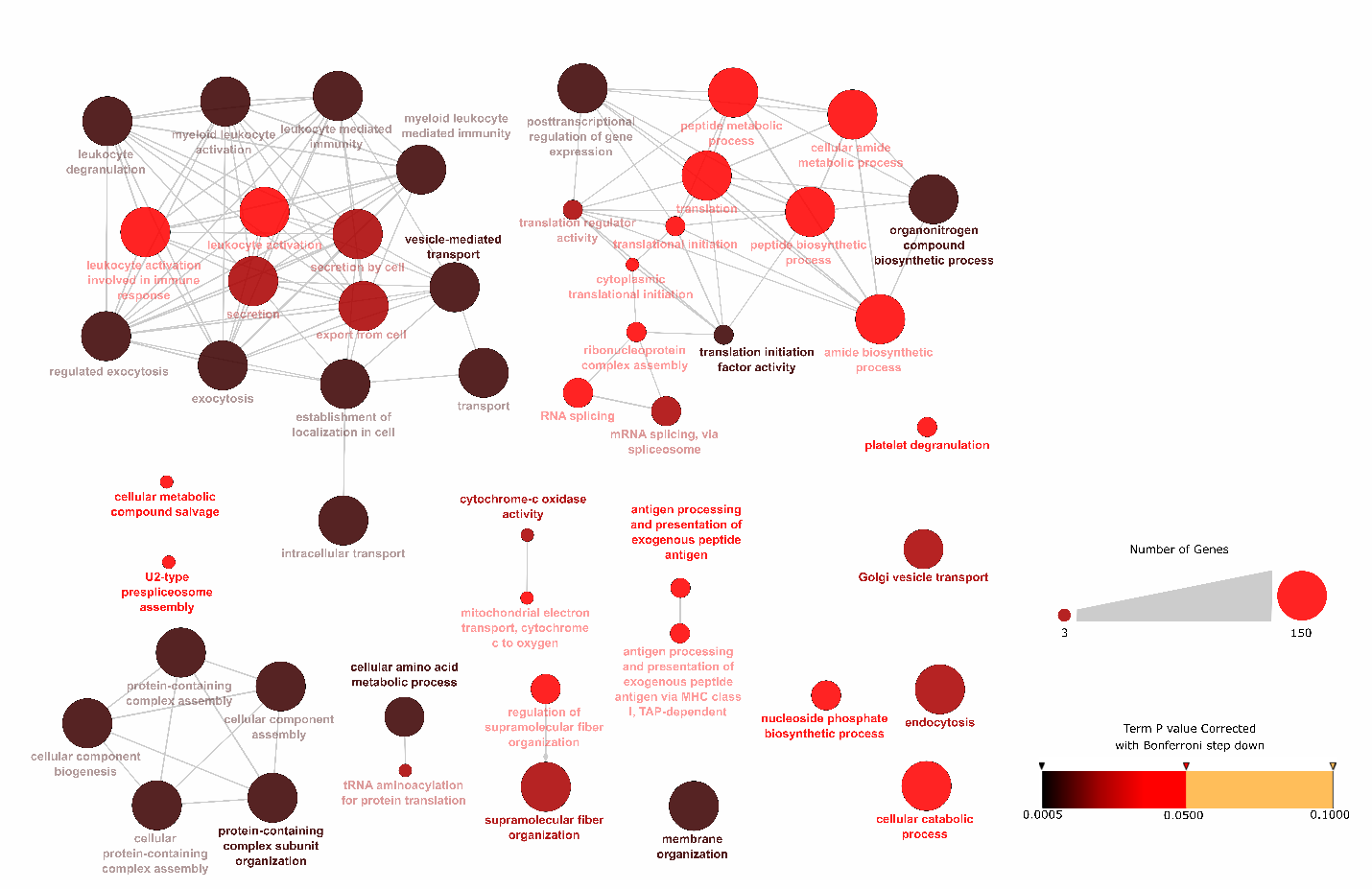


Supplemental Figure 4: ClueGO functional analysis of unique proteins identified in sample site brown. Node and text colour indicate the adjusted term p value. Node size indicates the number of proteins falling into the GO term.

In particular, proteins falling into the GO terms membrane organization, protein-containing complex and subunit organization, vesicle-mediated transport, organo-nitrogen compound biosynthetic process, and translation initiation factor activity are overrepresented. Most of the proteins unique to the brown sample site were filtered out for the analysis of both sub-anatomical region and sample site, as they did not meet the requirement of having been identified in at least 6 samples per sub-anatomical region.
