# Supplementary material for: A proteomic profile of the healthy human placenta
Source: Clin Proteomics. 2023 Jan 2;20:1. doi: 10.1186/s12014-022-09388-4 (PMC9808999; doi:10.1186/s12014-022-09388-4)
Supplement: Supplementary file 2 — Additional file 2. Table of DEPs between sub-anatomical regions. [file 12014_2022_9388_MOESM2_ESM.pdf]

		maternal vs fetal		maternal vs middle		middle vs fetal	
**Protein Ids**	**Gene names**	**adj. P-value**	**log2FC**	**adj. P-value**	**log2FC**	**adj. P-value**	**log2FC**
P08727	KRT19	0.000	-0.712	0.000	-0.784		
P62316	SNRPD2	0.001	-0.691			0.001	-0.665
Q13938	CAPS	0.030	-0.524				
Q15836	VAMP3	0.001	-0.523	0.001	-0.388		
P62140	PPP1CB	0.012	-0.471	0.012	-0.464		
P51692	STAT5B	0.000	-0.466	0.000	-0.381		
A0A0R4J2G3	NCEH1	0.000	-0.451			0.000	-0.301
A8K968	EPB41L3	0.003	-0.451				
O60749	SNX2	0.013	-0.442	0.013	-0.448		
Q7Z4H8	KDELC2	0.000	-0.430	0.000	-0.785	0.000	0.355
Q8TE68	EPS8L1	0.011	-0.423			0.011	-0.339
Q13630	TSTA3	0.012	-0.416	0.012	-0.452		
P07195	LDHB	0.000	-0.409	0.000	-0.324		
P21291	CSRP1	0.012	-0.406				
H0YJ34	FERMT2	0.005	-0.389	0.005	-0.315		
Q14980	NUMA1	0.025	-0.386	0.025	-0.427		
O00425	IGF2BP3	0.001	-0.362				
Q13492	PICALM	0.001	-0.358	0.001	-0.397		
Q13409	DYNC1I2	0.006	-0.355	0.006	-0.296		
Q9Y6U3	SCIN	0.000	-0.346	0.000	-1.150	0.000	0.805
P49207	RPL34	0.016	-0.345	0.016	-0.313		
P15924	DSP	0.000	-0.341	0.000	-0.369		
A0A286YFJ8	IGHG4	0.000	-0.334			0.000	-0.310
A0A0A0MSK5	TOR1AIP1	0.001	-0.331	0.001	-0.428		
Q9C0C2	TNKS1BP1	0.028	-0.330	0.028	-0.359		
P11940	PABPC1	0.005	-0.322	0.005	-0.287		
Q07960	ARHGAP1	0.000	-0.322	0.000	-0.413		
O60763	USO1	0.000	-0.321	0.000	-0.357		
P21128	ENDOU	0.017	-0.299	0.017	-0.260		
P08621	SNRNP70	0.028	-0.296	0.028	-0.290		
P05120	SERPINB2	0.015	-0.295				
Q9UNH7	SNX6	0.001	-0.295	0.001	-0.353		
Q9UQ72	PSG11	0.027	-0.294				
P31949	S100A11	0.004	-0.290				
P07384	CAPN1	0.003	-0.289	0.003	-0.440		
Q15717	ELAVL1	0.048	-0.280				
Q14974	KPNB1	0.000	-0.277	0.000	-0.315		
Q9BVK6	TMED9	0.034	-0.275				
G3XAM7	CTNNA1	0.027	-0.275	0.027	-0.255		
Q13151	HNRNPA0	0.001	-0.274			0.001	-0.205
P21980	TGM2	0.008	-0.270				
Q9UNM6	PSMD13	0.016	-0.269	0.016	-0.334		
P04792	HSPB1	0.003	-0.263	0.003	-0.194		
A0A1X7SBZ2	DDX17	0.000	-0.257	0.000	-0.144		
O14745	SLC9A3R1	0.000	-0.254	0.000	-0.229		
P12270	TPR	0.004	-0.252	0.004	-0.173		
Q00013	MPP1	0.000	-0.247	0.000	-0.164		
P05388	RPLP0	0.003	-0.247	0.003	-0.226		

P30622	CLIP1	0.008	-0.244			0.008	-0.223
P61247	RPS3A	0.000	-0.244	0.000	-0.232		
O95782	AP2A1	0.005	-0.243	0.005	-0.267		
Q96QR8	PURB	0.015	-0.240				
P23246	SFPQ	0.000	-0.235	0.000	-0.122		
Q15366	PCBP2	0.014	-0.222	0.014	-0.193		
P16403	HIST1H1C	0.023	-0.217				
Q15365	PCBP1	0.000	-0.217	0.000	-0.136		
Q9P2E9	RRBP1	0.000	-0.215	0.000	-0.115	0.000	-0.100
Q15056	EIF4H	0.000	-0.212			0.000	-0.220
O75396	SEC22B	0.000	-0.200	0.000	-0.224		
P14061	HSD17B1	0.045	-0.197				
Q15149	PLEC	0.000	-0.196	0.000	-0.171		
P05783	KRT18	0.000	-0.195	0.000	-0.316		
P30041	PRDX6	0.015	-0.195				
P02545	LMNA	0.001	-0.193	0.001	-0.199		
C9J0K6	SRI	0.000	-0.188	0.000	0.182	0.000	-0.370
Q71U36	TUBA1A	0.000	-0.187	0.000	-0.286		
Q96TA1	FAM129B	0.000	-0.187	0.000	-0.337	0.000	0.150
P31943	HNRNPH1	0.040	-0.186				
A0A087WT99	C11orf54	0.017	-0.185			0.017	-0.161
A6NLN1	PTBP1	0.001	-0.185	0.001	-0.170		
P23381	WARS	0.031	-0.181				
Q14204	DYNC1H1	0.014	-0.178	0.014	-0.210		
Q9Y3I0	RTCB	0.018	-0.178	0.018	-0.216		
P36871	PGM1	0.004	-0.177			0.004	-0.124
Q13576	IQGAP2	0.001	-0.176	0.001	-0.205		
P62851	RPS25	0.004	-0.176	0.004	-0.245		
Q14011	CIRBP	0.013	-0.171				
Q14103	HNRNPD	0.008	-0.171				
P60174	TPI1	0.005	-0.171				
Q14126	DSG2	0.000	-0.170	0.000	-0.324		
P07900	HSP90AA1	0.000	-0.170	0.000	-0.250		
O60506	SYNCRIP	0.043	-0.169				
O60664	PLIN3	0.000	-0.167	0.000	-0.148		
Q53EL6	PDCD4	0.011	-0.167	0.011	-0.155		
F8W6I7	HNRNPA1	0.023	-0.165				
A0A0X1KG75	COBLL1	0.001	-0.164	0.001	-0.155		
Q15075	EEA1	0.023	-0.158	0.023	-0.174		
Q5SSJ5	HP1BP3	0.020	-0.157	0.020	-0.166		
P08238	HSP90AB1	0.000	-0.157	0.000	-0.272	0.000	0.115
Q8WVM8	SCFD1	0.006	-0.157	0.006	-0.219		
A0A1B0GVD3	LIN28B	0.007	-0.156				
P55884	EIF3B	0.003	-0.156			0.003	-0.141
Q9UJ70	NAGK	0.000	-0.153	0.000	-0.168		
P04083	ANXA1	0.038	-0.151				
B0YIW6	ARCN1	0.000	-0.147	0.000	-0.163		
P60842	EIF4A1	0.000	-0.142	0.000	-0.231		
P21399	ACO1	0.000	-0.142	0.000	-0.275	0.000	0.134
Q9Y490	TLN1	0.002	-0.141	0.002	-0.093		

P40925	MDH1		0.001	-0.134					
Q15907	RAB11B		0.000	-0.133	0.000	-0.141			
Q8WUM4	PDCD6IP		0.001	-0.133	0.001	-0.173			
Q96HC4	PDLIM5		0.001	-0.131				0.001	-0.109
P67936	TPM4		0.005	-0.130				0.005	-0.125
Q15293	RCN1		0.001	-0.127				0.001	-0.150
Q00839	HNRNPU		0.032	-0.125					
B5MDF5	RAN		0.001	-0.124	0.001	-0.198			
Q13838	DDX39B		0.019	-0.120	0.019	-0.103			
Q13011	ECH1		0.000	-0.119	0.000	-0.238		0.000	0.119
P62820	RAB1A		0.031	-0.119	0.031	-0.128			
P30086	PEBP1		0.010	-0.115	0.010	-0.112			
P12429	ANXA3		0.005	-0.115	0.005	-0.144			
A0A3B3ISC6	COPA		0.020	-0.113	0.020	-0.119			
P54578	USP14		0.012	-0.110					
P37837	TALDO1		0.005	-0.110					
P46781	RPS9		0.003	-0.107	0.003	-0.141			
P63104	YWHAZ		0.005	-0.102	0.005	-0.087			
P00558	PGK1		0.005	-0.100	0.005	-0.137			
Q08211	DHX9		0.001	-0.096	0.001	-0.142			
Q14697	GANAB		0.000	-0.093	0.000	-0.200		0.000	0.107
P51149	RAB7A		0.000	-0.092	0.000	-0.126			
A0A087WYS1	UGP2		0.017	-0.089	0.017	-0.108			
P11142	HSPA8		0.012	-0.086	0.012	-0.079			
P13639	EEF2		0.003	-0.086	0.003	-0.068			
P28838	LAP3		0.000	0.108	0.000	0.391		0.000	-0.283
F8W1R7	MYL6		0.000	0.139	0.000	0.199			
P10599	TXN		0.005	0.141	0.005	0.201			
Q96CX2	KCTD12		0.005	0.145	0.005	0.148			
P69905	HBA1		0.002	0.164	0.002	0.209			
E9PKG6	NUCB2		0.000	0.167	0.000	0.308		0.000	-0.141
P01011	SERPINA3		0.003	0.171	0.003	0.225			
O75390	CS		0.002	0.174	0.002	0.139			
Q16557	PSG3		0.004	0.174	0.004	0.218			
P31146	CORO1A		0.004	0.176	0.004	0.232			
O60832	DKC1		0.001	0.190	0.001	0.159			
P12814	ACTN1		0.000	0.196	0.000	0.249			
P16152	CBR1		0.000	0.196	0.000	-0.175		0.000	0.371
C9K0U8	SSBP1		0.000	0.204	0.000	0.243			
Q9BPW8	NIPSNAP1		0.003	0.207	0.003	0.324			
P50454	SERPINH1		0.012	0.207				0.012	0.209
P30046	DDT		0.023	0.207					
P02749	APOH		0.016	0.209					
P11047	LAMC1		0.001	0.216	0.001	0.352			
P0DOY2	IGLC3		0.001	0.218	0.001	0.248			
P23284	PPIB		0.010	0.219					
P13716	ALAD		0.014	0.228				0.014	0.229
Q8NBS9	TXNDC5		0.015	0.233				0.015	0.188
P69892	HBG2		0.007	0.233	0.007	0.289			
P04217	A1BG		0.015	0.237					

A6NLM8	SSR4	0.026	0.247	0.026	0.230		
P19827	ITIH1	0.025	0.249	0.025	0.246		
P30043	BLVRB	0.002	0.251	0.002	0.279		
P02794	FTH1	0.004	0.251	0.004	0.368		
A0A1B0GW44	CTSD	0.000	0.254	0.000	0.312		
A0A0J9YXP8	GPI	0.017	0.255	0.017	0.294		
Q5T985	ITIH2	0.001	0.258	0.001	0.183		
P01871	IGHM	0.000	0.263	0.000	0.693	0.000	-0.430
A0A0G2JPR0	C4A	0.001	0.270	0.001	0.192		
P02008	HBZ	0.003	0.270	0.003	0.377		
P24043	LAMA2	0.031	0.273	0.031	0.287		
P61626	LYZ	0.007	0.283	0.007	0.319		
A0A286YEY1	IGHA1	0.000	0.291	0.000	-0.334	0.000	0.626
P35858	IGFALS	0.020	0.297				
P01031	C5	0.013	0.300				
P26447	S100A4	0.002	0.301	0.002	0.316		
Q96F07	CYFIP2	0.011	0.303			0.011	0.364
P06702	S100A9	0.006	0.307				
Q6NZI2	PTRF	0.005	0.311	0.005	0.301		
D6RFL4	CD14	0.007	0.313	0.007	0.444		
B7ZKJ8	ITIH4	0.001	0.314	0.001	0.226		
P02760	AMBP	0.002	0.323	0.002	0.237		
P50453	SERPINB9	0.000	0.330			0.000	0.235
P55268	LAMB2	0.000	0.330	0.000	0.388		
P23142	FBLN1	0.010	0.332	0.010	0.356		
P01857	IGHG1	0.000	0.332	0.000	0.497	0.000	-0.165
P21333	FLNA	0.000	0.336	0.000	0.576	0.000	-0.240
P01023	A2M	0.000	0.339	0.000	0.313		
O75131	CPNE3	0.013	0.341				
P00747	PLG	0.001	0.347	0.001	0.338		
P01024	C3	0.000	0.351			0.000	0.318
Q9NR12	PDLIM7	0.009	0.351	0.009	0.354		
Q99714	HSD17B10	0.002	0.353	0.002	0.323		
Q9NZM1	MYOF	0.000	0.360			0.000	0.386
P02679	FGG	0.004	0.362	0.004	0.272		
M0R0P1	FBL	0.002	0.370				
A0A087WUL2	PSMB3	0.017	0.379				
F2Z2V0	CPNE1	0.000	0.386			0.000	0.510
A6NKB8	RNPEP	0.001	0.386			0.001	0.211
P05109	S100A8	0.000	0.387			0.000	0.209
P04179	SOD2	0.011	0.387	0.011	0.490		
P08311	CTSG	0.015	0.388				
E9PIT3	F2	0.000	0.394	0.000	0.318		
P68032	ACTC1	0.000	0.396	0.000	0.505		
P02671	FGA	0.000	0.400	0.000	0.370		
Q00887	PSG9	0.000	0.402	0.000	0.154	0.000	0.248
P08603	CFH	0.000	0.403	0.000	0.281		
V9GYE3	APOA2	0.008	0.404	0.008	0.384		
H0YLF3	B2M	0.008	0.405	0.008	0.404		
P05164	MPO	0.001	0.408	0.001	0.265		

E9PFZ2	CP	0.000	0.413			0.000	0.253
P02675	FGB	0.001	0.416	0.001	0.377		
P01624	IGKV3D-15	0.044	0.417				
A0A286YEY4	IGHG2	0.000	0.429	0.000	0.257	0.000	0.172
P01834	IGKC	0.000	0.436	0.000	0.336		
P39060	COL18A1	0.000	0.437	0.000	0.268		
Q6ZN40	TPM1	0.046	0.444				
P27105	STOM	0.025	0.446				
A0A0B4J231	IGLL5	0.000	0.450	0.000	0.544		
F6WIT2	PPP2R4	0.001	0.457			0.001	0.347
Q5VT79	ANXA8L2	0.000	0.464			0.000	0.460
P02763	ORM1	0.000	0.472	0.000	0.229	0.000	0.243
A0A286YES1	IGHG3	0.000	0.494	0.000	0.447		
O94875	SORBS2	0.001	0.499	0.001	0.549		
A0A2Q2TTZ9	IGKV1-33	0.008	0.502	0.008	0.523		
Q08830	FGL1	0.006	0.502				
P69891	HBG1	0.001	0.503	0.001	0.607		
P0DP08	IGHV4-61	0.002	0.517	0.002	0.595		
A0A0D9SF54	SPTAN1	0.001	0.519	0.001	0.704		
P07360	C8G	0.002	0.519	0.002	0.366		
B4E1Z4	CFB	0.000	0.519	0.000	0.310	0.000	0.209
P04003	C4BPA	0.007	0.534				
A0A4W8ZXM2	IGHV3-72	0.000	0.539	0.000	0.285	0.000	0.254
P01619	IGKV3-20	0.000	0.560	0.000	0.443		
A0A087WUZ3	SPTBN1	0.003	0.561	0.003	0.635		
P02750	LRG1	0.000	0.609			0.000	0.419
F8W1A4	AK2	0.009	0.619				
A0A0A0MSV6	C1QB	0.012	0.642	0.012	0.581		
P21810	BGN	0.000	0.673	0.000	0.732		
P08246	ELANE	0.000	0.774	0.000	0.559		
A0A087X0K0	COL15A1	0.000	0.813	0.000	0.653		
P27487	DPP4	0.000	0.823	0.000	0.679		
E7EQB2	LTF	0.000	0.837			0.000	0.685
P20742	PZP	0.000	0.886	0.000	-1.407	0.000	2.292
B1AKG0	CFHR1	0.000	0.972	0.000	0.797		
Q13103	SPP2	0.000	0.976	0.000	0.775		
P01780	IGHV3-21	0.000	0.987	0.000	0.527		
P61970	NUTF2	0.016	0.998	0.016	1.011		
Q05707	COL14A1	0.000	1.159	0.000	0.573	0.000	0.586
P35749	MYH11	0.000	1.238	0.000	1.476		
A0A0A0MSD0	SVEP1			0.017	-0.445	0.017	0.372
B1AHL2	FBLN1			0.000	0.000		
Q5QPL9	RALY			0.042	-0.429		
P49458	SRP9			0.031	-0.427		
P04114	APOB			0.001	-0.425		
Q9NZN4	EHD2			0.023	-0.406		
Q9NPH2	ISYNA1			0.000	-0.396	0.000	0.394
P50570	DNM2			0.042	-0.385		
P02786	TFRC			0.000	-0.368	0.000	0.293
P22234	PAICS			0.027	-0.359		

P30533	LRPAP1		0.000	-0.353	0.000	0.225
Q13200	PSMD2		0.010	-0.347		
O43399	TPD52L2		0.010	-0.333		
Q5JXI8	FHL1		0.012	-0.329		
Q9Y2V2	CARHSP1		0.016	-0.328		
Q8NC56	LEMD2		0.001	-0.321		
A0A024R571	EHD1		0.001	-0.304		
M0R210	RPS16		0.000	-0.304	0.000	0.213
P33176	KIF5B		0.048	-0.303		
P00966	ASS1		0.001	-0.288	0.001	0.320
P05187	ALPP		0.003	-0.288	0.003	0.231
Q9NSD9	FARSB		0.027	-0.284		
P00738	HP		0.006	-0.278		
P35998	PSMC2		0.020	-0.275		
P00387	CYB5R3		0.014	-0.274		
Q01469	FABP5		0.000	-0.261	0.000	0.270
O60493	SNX3		0.008	-0.255		
P30038	ALDH4A1		0.005	-0.254		
A0A0A0MSW4	PITPNB		0.016	-0.249		
H3BLU7	AKR7A2		0.000	-0.240	0.000	0.221
P46940	IQGAP1		0.000	-0.239	0.000	0.179
Q06210	GFPT1		0.003	-0.238		
R4GNH3	PSMC3		0.016	-0.233		
P49411	TUFM		0.011	-0.230		
A0A0C4DGG1	PACSIN3		0.026	-0.226		
P48735	IDH2		0.000	-0.218	0.000	0.199
Q5JP53	TUBB		0.009	-0.208		
H0YFD6	HADHA		0.005	-0.203		
P08670	VIM		0.005	-0.201		
P02787	TF		0.001	-0.201	0.001	0.154
P30153	PPP2R1A		0.001	-0.200		
P55084	HADHB		0.005	-0.198		
A0A087X0X3	HNRNPM		0.004	-0.195	0.004	0.125
O95833	CLIC3		0.027	-0.191		
P68371	TUBB4B		0.022	-0.187		
A0A087WY71	AP2M1		0.003	-0.182		
P15121	AKR1B1		0.012	-0.177		
P62266	RPS23		0.018	-0.177		
P61106	RAB14		0.019	-0.176		
Q9BS26	ERP44		0.000	-0.172	0.000	0.219
O00203	AP3B1		0.046	-0.169		
A0A087X054	HYOU1		0.019	-0.158		
O75874	IDH1		0.017	-0.153	0.017	0.140
P14625	HSP90B1		0.012	-0.153		
Q16555	DPYSL2		0.016	-0.148		
A0A0U1RQF0	FASN		0.045	-0.145		
E7EQR4	EZR		0.012	-0.144		
A0A0A0MR02	VDAC2		0.031	-0.140		
P12956	XRCC6		0.003	-0.139	0.003	0.116
M0R0F0	RPS5		0.050	-0.135		

P20700	LMNB1		0.015	-0.129		
P21964	COMT		0.023	-0.121		
P10809	HSPD1		0.017	-0.121		
P30101	PDIA3		0.023	-0.118		
P52209	PGD		0.035	-0.111	0.035	0.116
P24534	EEF1B2		0.040	-0.110		
P11021	HSPA5		0.027	-0.105		
P08758	ANXA5		0.013	-0.074	0.013	0.089
P53396	ACLY		0.018	0.087		
P39019	RPS19		0.015	0.102	0.015	-0.113
P62879	GNB2		0.028	0.106		
P07954	FH		0.020	0.123		
P59998	ARPC4		0.011	0.126		
P62857	RPS28		0.038	0.127		
P09382	LGALS1		0.026	0.136	0.026	-0.151
O95865	DDAH2		0.011	0.139	0.011	-0.164
P62424	RPL7A		0.001	0.149	0.001	-0.157
P41091	EIF2S3		0.013	0.154		
P06756	ITGAV		0.031	0.157		
P62979	RPS27A		0.011	0.162		
A0A087WY82	F11R		0.022	0.164		
A0A2R8YCS7	SAMHD1		0.034	0.166		
Q01105	SET		0.005	0.179		
P25311	AZGP1		0.031	0.193		
P46783	RPS10		0.018	0.193	0.018	-0.183
P52597	HNRNPF		0.046	0.200		
P00505	GOT2		0.025	0.207		
S4R3H4	ACIN1		0.027	0.220		
P35579	MYH9		0.002	0.223		
Q9BW30	TPPP3		0.001	0.224	0.001	-0.268
O75369	FLNB		0.034	0.225		
Q9Y224	C14orf166		0.006	0.247		
E9PDI4	LAD1		0.001	0.257	0.001	-0.224
Q9HDC9	APMAP		0.000	0.270	0.000	-0.372
E7ER27	HSD17B4		0.017	0.291		
A0A087X0K9	TJP1		0.003	0.305		
P01008	SERPINC1		0.038	0.309		
P07942	LAMB1		0.018	0.333		
P14543	NID1		0.001	0.359	0.001	-0.218
Q9NZ08	ERAP1		0.000	0.365	0.000	-0.456
Q01995	TAGLN		0.014	0.387	0.014	-0.333
Q15582	TGFBI		0.027	0.392		
P11464	PSG1		0.000	0.411	0.000	-0.305
Q9NZD4	AHSP		0.010	0.430		
A0A0A0MTC7	LAMA4		0.026	0.430		
P02775	PPBP		0.001	0.432	0.001	-0.361
P06727	APOA4		0.008	0.435		
M0R0B3	PSG4		0.015	0.439		
Q14019	COTL1		0.000	0.444	0.000	-0.287
P07585	DCN		0.010	0.461		

Q96HE7	ERO1L			0.012	0.469		
A0A0B4J2A4	ACAA2			0.015	0.470		
E5RGU4	EIF3H			0.013	0.504		
P02792	FTL			0.048	0.633		
P17661	DES			0.000	0.794	0.000	-0.743
Q14847	LASP1					0.010	-0.216
B8ZZQ6	PTMA					0.009	-0.216
P27695	APEX1					0.033	-0.214
P23526	AHCY					0.017	-0.159
Q8NC51	SERBP1					0.026	-0.121
Q16658	FSCN1					0.012	-0.106
P30044	PRDX5					0.032	0.107
P54922	ADPRH					0.038	0.131
P36957	DLST					0.039	0.136
P60866	RPS20					0.022	0.147
P09525	ANXA4					0.001	0.176
P08559	PDHA1					0.020	0.205
P12532	CKMT1A					0.013	0.228
O60829	PAGE4					0.012	0.266
G3V1D3	DPP3					0.050	0.291
Q9Y2D5	AKAP2					0.035	0.344
A0A1B0GTB8	CPT2					0.004	0.345
P59666	DEFA3					0.000	0.424
O43681	ASNA1					0.031	0.450
P15090	FABP4					0.022	0.466
P46778	RPL21					0.016	0.515
